# Portable and affordable cold air plasma source with optimized bactericidal effect

**DOI:** 10.1038/s41598-024-66017-w

**Published:** 2024-07-10

**Authors:** Myron Klenivskyi, Josef Khun, Laura Thonová, Eva Vaňková, Vladimír Scholtz

**Affiliations:** 1 Department of Physics and Measurements, University of Chemistry and Technology, Prague, Czech Republic; 2 Department of Physics, Faculty of Nuclear Sciences and Physical Engineering, Czech Technical University in Prague, Prague, Czech Republic

**Keywords:** Corona discharge, Ion wind, Cold air plasma, Bactericidal effect, Reactive oxygen and nitrogen species, Microbiology, Engineering, Physics

## Abstract

The paper reports a low-cost handheld source of a cold air plasma intended for biomedical applications that can be made by anyone (detailed technical information and a step-by-step guide for creating the NTP source are provided). The plasma source employs a 1.4 W corona discharge in the needle-to-cone electrode configuration and is an extremely simple device, consisting basically of two electrodes and a cheap power supply. To achieve the best bactericidal effect, the plasma source has been optimized on *Escherichia coli*. The bactericidal ability of the plasma source was further tested on a wide range of microorganisms: *Staphylococcus aureus* as a representative of gram-positive bacteria, *Pseudomonas aeruginosa* as gram-negative bacteria, *Candida albicans* as yeasts, *Trichophyton interdigitale* as microfungi, and *Deinococcus radiodurans* as a representative of extremophilic bacteria resistant to many DNA-damaging agents, including ultraviolet and ionizing radiation. The testing showed that the plasma source inactivates all the microorganisms tested in several minutes (up to 10^5^–10^7^ CFU depending on a microorganism), proving its effectiveness against a wide spectrum of pathogens, in particular microfungi, yeasts, gram-positive and gram-negative bacteria. Studies of long-lived reactive species such as ozone, nitrogen oxides, hydrogen peroxide, nitrite, and nitrate revealed a strong correlation between ozone and the bactericidal effect, indicating that the bactericidal effect should generally be attributed to reactive oxygen species. This is the first comprehensive study of the bactericidal effect of a corona discharge in air and the formation of long-lived reactive species by the discharge, depending on both the interelectrode distance and the discharge current.

## Introduction

A non-thermal plasma (NTP) is an ionized gaseous medium containing highly reactive species that give it special, sought-after properties, such as bactericidal. Particularly significant is that a NTP can eradicate pathogens from delicate items without damaging them^1^. NTP is rapidly gaining popularity thanks to the great prospects for its use in medicine, biology, ecology, industry, and agriculture^2–11^. The demand and promise of a NTP is confirmed by a large number of articles published annually. The areas of application of a NTP are being actively explored and are constantly expanding.

Especially promising is the use of a NTP in medicine^12–17^. NTP has shown its effectiveness in disinfection^18–20^, wound healing^21–26^, tumor suppression^27–31^, treatment of skin diseases^32–35^, oral/dental diseases^36–39^, nail fungus^40,41^, etc. Of particular note is that a NTP can inhibit the growth of biofilms and even antibiotic-resistant bacteria^42–45^. Moreover, experiments show no development of bacterial resistance to plasma treatment^46–50^. Due to the fact that pathogens have been demonstrated to develop resistance to antibiotics^51–57^, the study and development of NTP sources as an alternative to traditional drug therapy is of particular importance.

One of the simplest ways to generate a NTP is to ignite an electric discharge in the air. A plasma generated in the air is rich in reactive oxygen species (ROS) and reactive nitrogen species (RNS), which endow the plasma with bactericidal properties^46,58–60^. In fact, a discharge in the air produces quite complex reactive chemistry^61–64^. The composition and content of plasma species largely depend on a discharge that generates the plasma. There are various discharges used to produce a NTP^65–67^. Among the most commonly used are atmospheric pressure plasma jets^68–70^, dielectric barrier discharges^71,72^, resistive barrier discharges^73^, DC-driven discharges^74,75^, gliding arc discharges^76^.

To date, there are a great many articles reporting various NTP sources but most of them are experimental setups that are not adapted for practical use. In fact, most NTP sources used in research are bulky, non-portable, and expensive. Usually, the most expensive part of a plasma source is the high-voltage power supply. However, of particular importance is not only to achieve good results in a laboratory but also to make them available for wide practical use. Due to the high cost of power supplies, NTP sources used for research are impractical in practice. Fortunately, generating a NTP does not require much energy since the high energy deposited per unit volume of a plasma causes it to heat up. A high-voltage power supply with a mean power of several watts is quite enough to generate a NTP^61,77^. Commercially available DC high-voltage power supplies of low-power are cheap and compact enough to allow the development of a portable and affordable NTP source. The aim of this research was precisely to develop a cheap, handheld, and efficient NTP source for biomedical applications.

It is worth noting that few attempts to create portable NTP sources have been made (e.g.^78–84^), but the reported plasma sources use barrier discharges and atmospheric pressure plasma jets, while other types of discharge have not received due attention. However, the use of DC discharges makes it possible to develop much simpler and cheaper NTP sources. This is because an electrode system for a DC discharge is simpler, and DC high-voltage power supplies are cheaper. In addition, DC discharges require no additional measures to transfer active species to the object being processed, which is one of their main advantages. A strong ion wind induced by a DC discharge effectively copes with this task. Thus, there is no need to use any compressed gas or pumping system to create a flow that transfers active species to a treatment object. Note that the term “ion wind” refers to a flow of air resulting from the transfer of momentum to neutral air molecules when colliding with ions accelerated by an electric field.

Unfortunately, surface barrier discharges have a low mass-transfer of plasma species so the treatment object must be in contact with the discharge. This makes it difficult to process objects of complex geometry, in particular, with holes or with a concave/convex surface. Ni et al.^78^ managed to create a flow of plasma species directed perpendicularly from the surface barrier discharge using a special geometry of the electrodes. Unfortunately, an increase in the flow speed is accompanied by a decrease in the effective area of the surface barrier discharge, which negatively affects the efficiency of the NTP source. As for a volume dielectric barrier discharge, it has a fixed discharge gap, which imposes restrictions on the size of objects being processed. Moreover, an object introduced into the discharge gap can affect the discharge operation.

As far as atmospheric pressure plasma jets are concerned, they are free from the above disadvantages and can be used to process objects of arbitrary geometry. However, atmospheric pressure plasma jets require a gas supply usually provided by cylinders with compressed feed gases^81^ or a system pumping the air through the discharge^79,82^. This makes such NTP sources more complex, expensive, and less portable.

It should be noted that we reported two portable NTP sources based on DC discharges^85^. The first plasma source uses a so-called cometary discharge^86,87^ formed in a needle-to-needle electrode configuration. Despite the advantages and well-pronounced bactericidal effect^85–93^, the cometary discharge is not stable enough and can switch to another discharge mode. Because of this, the discharge requires constant monitoring and adjustment, which make it difficult to use this NTP source in practice.

In contrast to the plasma source based on the cometary discharge, the second one uses a corona discharge formed in a needle-to-cone electrode configuration and is highly reliable in operation^85^. The conical electrode makes it easy to transfer plasma species through the electrode to the treatment object by means of the ion wind induced by the discharge. In fact, the second NTP source is extremely simple and it is just a corona discharge and a high-voltage power supply placed in a handheld plastic case. However, the development of not just a portable and affordable, but at the same time an efficient NTP source has not been given due attention, which is what this research is dedicated to.

Thus, the research aims to develop a simple, handheld, and low-cost NTP source based on a corona discharge in air with an optimized bactericidal effect. For this purpose, a comprehensive study of the bactericidal and physical properties of a corona discharge in air is carried out. The bactericidal and physical properties of the discharge, as well as the formation of various reactive species by the discharge, are studied depending on two parameters: the interelectrode distance and the discharge current. In addition, the bactericidal ability of the optimized discharge is tested on a wide range of pathogens, including microfungi, yeasts, gram-positive and gram-negative bacteria.

## Methods and techniques

### Description of the NTP source

The NTP source presented in this paper is based on a DC discharge in the needle-to-cone electrode configuration chosen for its advantageous features. One of them is that plasma species are blown out of the conical electrode in a narrow flow thanks to the ion wind induced by the discharge. This feature makes it simple to treat objects of interest just placing them under the conical electrode and at the same time provides a well-pronounced bactericidal effect. Note that a treatment object is placed outside the electrode system, so it does not affect the discharge operation.

A DC discharge usually requires a ballast resistor to be connected in series with the discharge to prevent it from arcing and damaging the power supply. Fortunately, low-cost DC high-voltage power supplies of low-power have high output impedance, so additional ballast resistance can be omitted. The absence of the need for a ballast resistor makes the plasma source an extremely simple device, essentially consisting of two electrodes and a power supply.

The power supply of the NTP source comprises a step-up high-voltage DC/DC converter and a 12 V voltage source. The electrode system consists of a conical electrode and a needle (a Medoject 0.6 mm × 25 mm intramuscular injection needle). The conical electrode was made of brass. It is approximately 11 mm in diameter at the top and 8 mm at the bottom. The electrode system is connected directly to the output of the high-voltage DC/DC converter without a ballast resistor. The needle electrode is connected to the negative terminal of the high-voltage power supply and the conical electrode is connected to the positive one. Detailed technical information and a step-by-step guide for creating the NTP source are available on our website^94^.

### Methods for studying the bactericidal properties of the discharge

**Figure 1:**
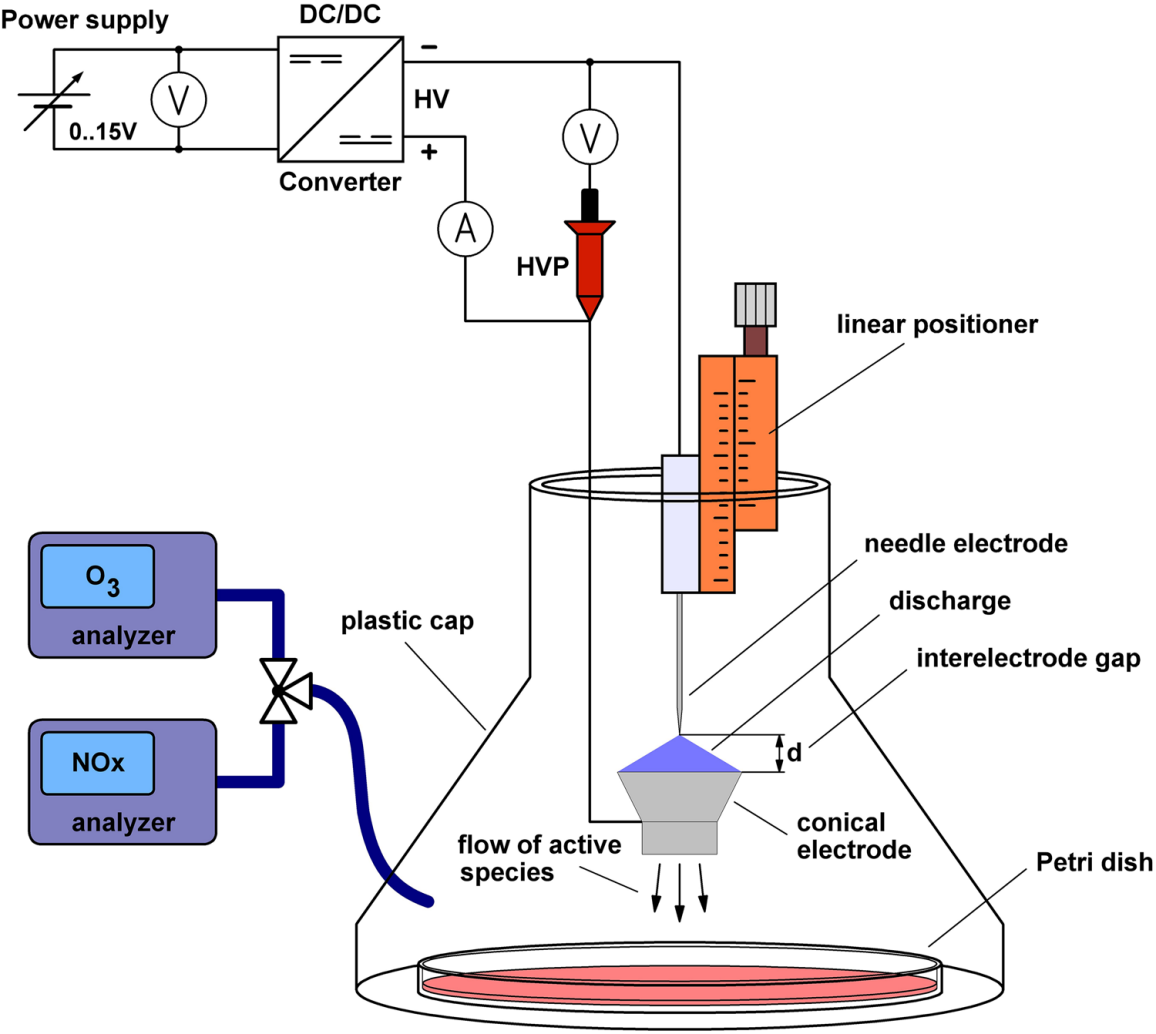
Experimental setup for studying the bactericidal and physical properties of the discharge.

To study the properties of the discharge and optimize it for the best bactericidal effect, we used the experimental setup shown schematically in Fig. 1. To regulate the high-voltage across the electrodes, we powered the high-voltage DC/DC converter from a regulated DC low-voltage source. In addition, the needle electrode was mounted on a micrometric linear positioner so that the interelectrode distance could be accurately set.

The bactericidal properties of the discharge were investigated based on its effect on *Escherichia coli*. A Petri dish containing Mueller–Hinton agar was inoculated with the bacteria and placed approximately 1 cm below the conical electrode to expose to the discharge. To study the bactericidal effect of the discharge over a wide range, we used Petri dishes containing 10^5^, 10^6^, 10^7^, and 10^8^ colony-forming units (CFUs) of *E. coli*. After exposure to the discharge for 90 s, the Petri dishes were kept at 37 °C in an incubator for 14 h to cultivate the bacteria. The bactericidal effect of the discharge was quantified as the number of inactivated CFUs, estimated from the area of the inhibition zone. Since plasma species are blown out of the conical electrode in a narrow flow, they form an inhibition zone, the area of which varies slightly with changes in the discharge parameters, making it difficult to study the bactericidal effect of the discharge. In order to overcome this, the discharge together with a Petri dish were covered with a 3D-printed plastic cap (Fig. 1), which, by accumulating active species, evens out the bactericidal effect over the Petri dish area. Note that the discharge was not completely closed since the cap had a hole in the top for adjusting the electrodes.

### Methods for studying the physical properties of the discharge

The electrical characteristics of the discharge were measured using two UT804 UNI-T digital multimeters (Uni-Trend Technology Co., China). The discharge current was measured directly by the multimeter, while the discharge voltage was measured through a 1000:1 Pintek HVP-40 high-voltage probe (Pintek Electronics Co., New Taipei, Taiwan). Emission spectra were recorded with a resolution of less than 1 nm using an HR2000+ spectrometer (Ocean Optics, Ostfildern, Germany). The radiation from the discharge was supplied to the spectrometer using a P200-2-UV–Vis optical fiber (Ocean Optics, Ostfildern, Germany).

An analytical balance ABS 220-4N (Kern, Balingen, Germany) was used to study the influence of the discharge current and the interelectrode distance on the speed of the ion wind induced by the discharge. The platform of the balance was approximately 3 cm below the conical electrode. The dependences obtained in relative units were subsequently normalized to absolute values. The absolute speed of the ion wind was measured using a multifunction measuring tool Testo 400 (Testo, Prague, Czech Republic) with a hot-wire probe 074. Measurements using the balance simplified data acquisition and its processing since it was connected directly to a PC via the RS-232 interface. In addition, the balance is less sensitive to fluctuations in speed of ion wind and temperature, which affect the quality of measurements. The temperature of species blown out by the discharge was also measured using Testo 400 with the probe 074 comprising a thermal sensor (NTC thermistor). The probe was placed approximately 1 cm below the conical electrode.

Nitrogen oxides were measured using a Serinus 40H NO_x_ Analyzer (ACOEM Ecotech, Melbourne, Australia), which is based on chemiluminescent detection. Ozone concentration was measured by means of a UV-100 ozone analyzer (Eco Sensors, Santa Fe, USA). Hydrogen peroxide was detected using Quantofix Peroxide 100 test strips (Macherey–Nagel, Düren, Germany), nitrate and nitrite were measured using Quantofix Nitrate/Nitrite test strips (Macherey–Nagel, Düren, Germany), and pH was controlled using Lach:ner test strips (Lach-Ner, Neratovice, Czech Republic). Measurements of ozone and nitrogen oxides were taken at 90 s after turning on the discharge to have similar time conditions as in the measurements of the bactericidal properties of the discharge. As for the measurements with the test strips, they were exposed to the discharge for 3 min to obtain reliable readings within their measurement range. Semi-quantitative measurements with the test strips were employed only to map the behavior of the formation of the reactive species and reveal their possible correlation with the bactericidal effect of the discharge.

### Cultivation of microorganisms for testing the NTP source

When testing the bactericidal ability of the developed NTP source on various microorganisms, their cultivation conditions were as follows. *Pseudomonas aeruginosa* (PAO1) and *Staphylococcus aureus* (wild-type strain) were cultivated on Mueller–Hinton agar for 24 h at 37 °C. *Candida albicans* (strain SC5314/ATCC MYA-2876) was cultivated on Sabouraud dextrose agar for 24 h at 37 °C. *Trichophyton interdigitale* 6603 (a clinical isolate provided by Laboratory of Clinical Mycology, Public Health Institute in Ostrava) was cultivated on Sabouraud dextrose agar for 72 h at 30 °C. *Deinococcus radiodurans* (CCM 1700) was cultivated on LB-agar for 96 h at 30 °C.

## Results and discussion

A critical part of developing an effective NTP source is optimizing the discharge. To determine the optimal discharge conditions that provide the best bactericidal effect of a plasma source, it is necessary to study the bactericidal and electrical properties of the discharge. Of importance is also the study of other physical properties of the discharge to provide insight into the operation of the NTP source. Once the discharge optimization is complete, the next step is to develop the design of the plasma source and test the bactericidal ability of the developed NTP source on different microorganisms. The following sections provide a detailed description of each of the steps taken.

### Bactericidal properties of the discharge

In order to develop an efficient plasma source, the discharge, underlying its operation, must be properly optimized. For the chosen electrode configuration, it is required to determine only the optimal discharge current and interelectrode distance, at which the discharge has the maximum bactericidal effect. This was done by studying the effect of the discharge on *E. coli* since this microorganism is convenient and widely used in research. We assume that the discharge parameters under which the maximum bactericidal effect on *E. coli* is achieved will also be optimal for most other microorganisms.

**Figure 2:**
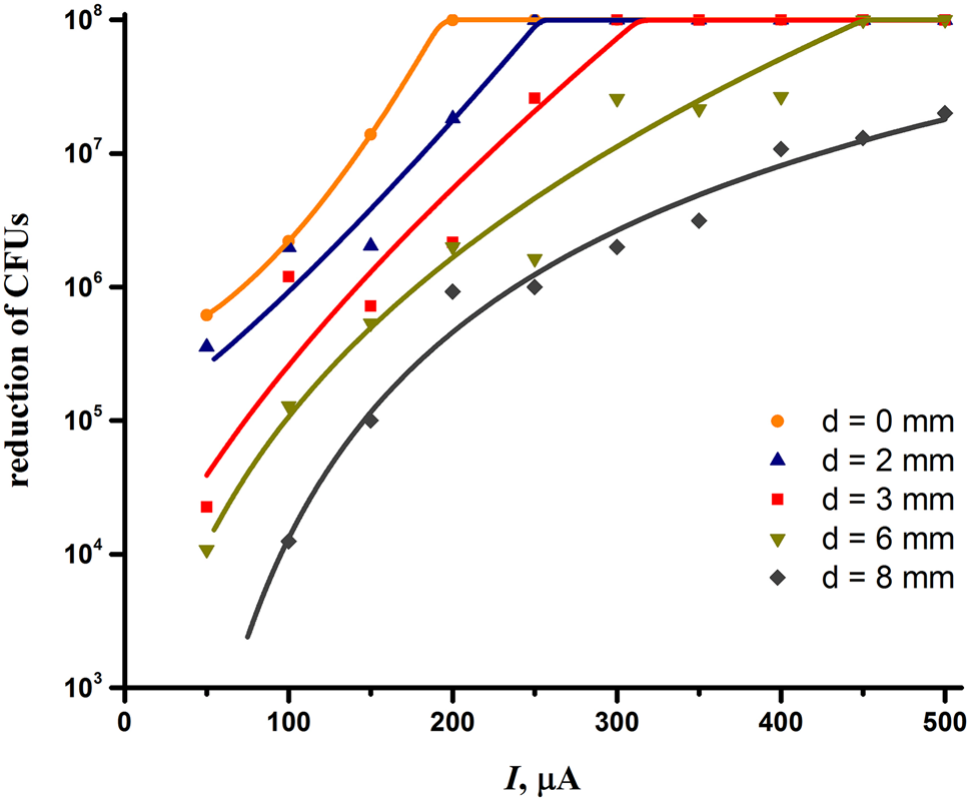
Decrease in CFUs of *E. coli* when exposed to the discharge for 90 s, depending on the discharge current and interelectrode distance.

The inactivating effect of the discharge on *E. coli* when exposed for 90 s is presented in Fig. 2. The graph shows the reduction of *E. coli* CFUs as a function of the discharge current and interelectrode distance. As can be seen, the bactericidal effect of the discharge increases significantly with increasing the discharge current. Note that the achieved maximum inactivation of *E. coli* equal to 10^8^ CFUs is not due to the limited bactericidal effect of the discharge but to the number of CFUs inoculated into a Petri dish.

On the other hand, the inactivating effect of the discharge also depends on the distance between the electrodes, and the shorter the interelectrode distance the higher the effect is (Fig. 2). As follows from the dependences, the maximum bactericidal effect is achieved at the interelectrode distance equal to 0 mm, i.e., when the tip of the needle reaches the plane of the conical electrode. It is worth noting that we also measured the inactivation of *E. coli* at negative interelectrode distances by inserting the tip of the needle as far as 3 mm into the conical electrode. However, the inactivating effect of the discharge with the needle inside the conical electrode turned out to be virtually the same as in the case of the interelectrode distance equal to 0 mm.

Thus, based on the conditions for the maximum inactivation of *E. coli*, one can choose the optimal distance between the electrodes equal to 0 mm and the discharge current as high as possible. However, the maximum discharge current can be limited by the power of a high-voltage power supply and the risk of the discharge switching to another mode. In order to choose the right discharge parameters, it is also necessary to study the electrical characteristics of the discharge.

### Physical properties of the discharge

Since the bactericidal properties of the discharge were studied in the previous section, the next step is to explore the physical properties of the discharge. On the one hand, a study of the electrical characteristics of the discharge is necessary to choose proper discharge parameters for the NTP source. On the other hand, it is of interest to diagnose the NTP and reactive species produced by the discharge since they determine the operation of the NTP source. Moreover, a comprehensive study of the physical properties of a corona discharge in air, along with its bactericidal properties, is of great scientific value.

**Figure 3:**
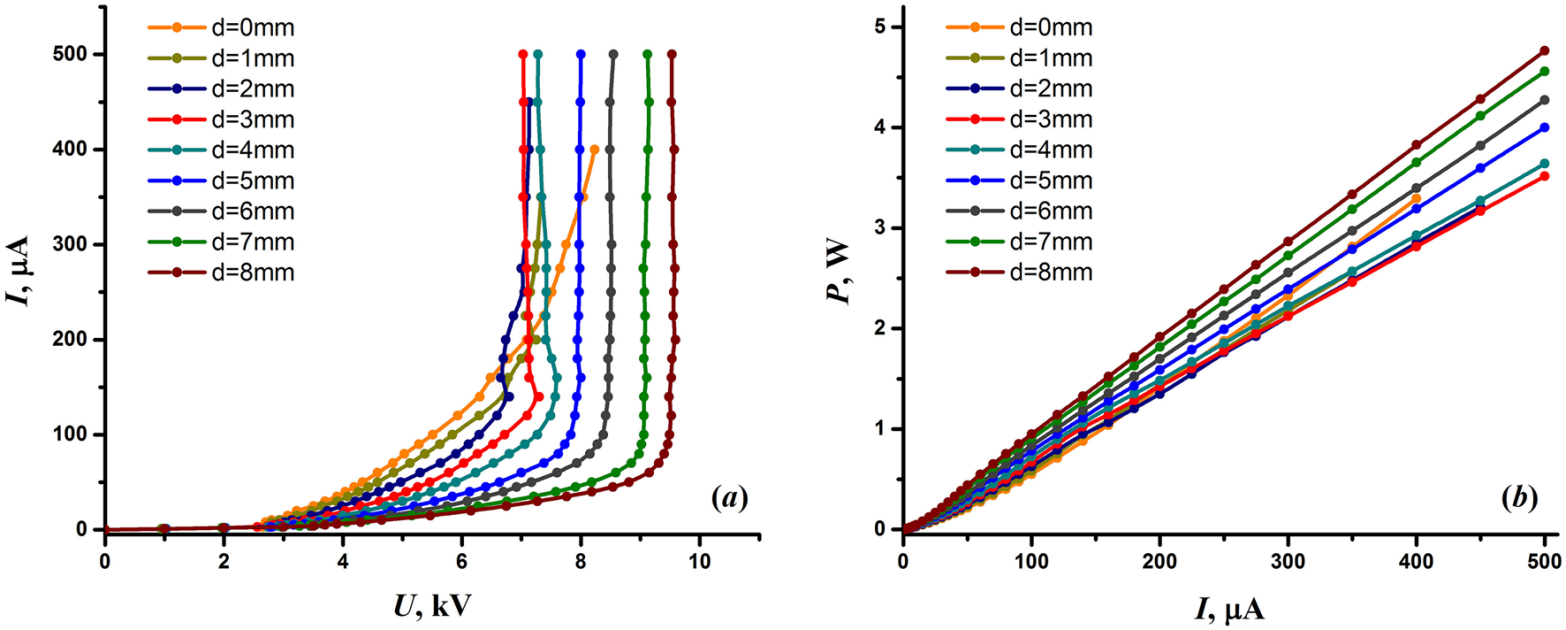
Volt-ampere characteristics of the discharge (**a**) and dependences of the discharge power on the discharge current (**b**) at different interelectrode distances.

Figure 3a shows volt–ampere characteristics of the discharge at different interelectrode distances. The characteristics were measured by varying the discharge voltage, which is controlled by the input voltage of the high-voltage DC/DC converter (Fig. 1). The discharge modes were determined from the measured volt–ampere characteristics and the visual appearance of the discharge. As one can see in Fig. 3a, the volt–ampere characteristics consist of two parts corresponding to different modes of the corona discharge. The first relatively flat part belongs to the Trichel pulse corona mode, and the second steep part to the steady glow corona mode, which gradually transitions to the streamer mode at higher discharge currents. In the streamer corona mode, the development of streamers from the edge of the conical electrode was observed. An increase in the discharge current led to a growth of the streamers towards the needle electrode and upon reaching a certain critical value (usually above 500 μA), the discharge began to be accompanied by random sparks. The spark mode was considered undesirable and was avoided in the research. With a decrease in the interelectrode distance, the sparks appeared at lower discharge currents. It should also be noted and taken into account that an increase in humidity lowers the discharge current at which sparks occur.

One can notice in Fig. 3a that the shape of the volt-ampere characteristics at large interelectrode distances was similar but changed at short distances. This seems due to the fact that at large interelectrode distances, the discharge was established between the needle and the edge of the conical electrode, while with a decrease in the interelectrode distance, the internal wall of the conical electrode was more and more involved in the establishment of the discharge. Similar effects were noted in work^95^. It is worth also noting that the discharge current, corresponding to the inflection of a volt-ampere characteristic, shifted towards higher values with a decrease in the interelectrode distance (Fig. 3a). Since the discharge voltage changes very little in the steady glow corona mode, we had to use the discharge current as the main parameter of the discharge when studying its various characteristics.

Due to the limited mean power of cheap high-voltage power supplies, the power deposited into the discharge is of high importance. Figure 3b shows how the power deposited into the discharge depends on the discharge current and interelectrode distance. As can be seen, the discharge power had an almost linear dependence on the discharge current. It should be noted, however, that the discharge power has a parabolic dependence in the Trichel pulse corona mode, i.e., at discharge currents below 50–100 μA, depending on the interelectrode distance.

One could expect that the bactericidal properties of the discharge would directly depend on the power deposited into the discharge. Although it looked like this for a fixed interelectrode distance, in general, larger interelectrode distances required higher power to achieve the same inactivating effect (Fig. 2). For example, the power of 3.8 W was required to achieve an *E. coli* inactivation of 10^8^ CFUs at the interelectrode distance of 6 mm and 1.4 W at the distance of 0 mm. In truth, the bactericidal effect of the discharge was the lowest at the largest interelectrode distances despite the fact that the power deposited into the discharge was maximum. Thus, maximum power is not a criterion for determining optimal discharge parameters that provide the best bactericidal effect.

Taking into account the bactericidal and electrical characteristics of the discharge, we have chosen the following parameters of the discharge for the NTP source: the interelectrode distance of 0 mm and the discharge current of 200 μA. The discharge current has been taken with a margin to ensure reliable operation of the discharge. A higher discharge current is not recommended at the interelectrode distance of 0 mm due to the fact that high humidity can cause single sparks at the discharge current of approximately 250 μA. Under the chosen conditions, the power deposited into the discharge is 1.4 W. Therefore, a cheap high-voltage power supply is sufficient to create an efficient NTP source for biomedical applications.

It could be of interest to mention that the discharge parameters in the initial non-optimized version of the NTP source were as follows: the interelectrode distance was approximately 3.3 mm and the discharge current was 150 μA^85^. One can see in Fig. 2 that these conditions correspond to the inactivation of *E. coli* approximately two orders of magnitude lower than at *d* = 0 mm and *I* = 200 μA. Thus, the NTP source being developed is expected to have a significantly enhanced inactivating effect compared to the original non-optimized plasma source.

One of the important parameters of a plasma is its temperature. For NTP sources used in biomedical applications, the temperature of a plasma (the temperature of species that are in contact with a treatment object) is of fundamental importance since a relatively hot plasma can cause thermal damage to living tissues.

**Figure 4:**
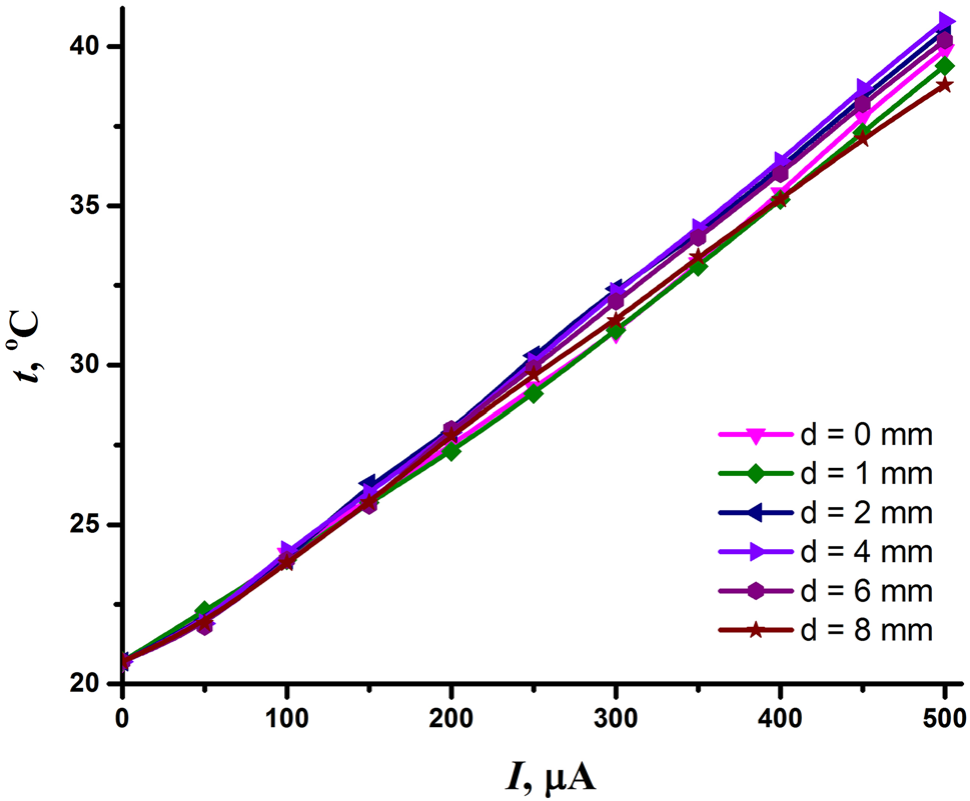
Temperature of the air flow at the outlet of the conical electrode, depending on the discharge current and interelectrode distance.

To clarify this issue, we measured the temperature of the air flow at the outlet of the conical electrode. The temperature dependence of the air flow on the discharge current and interelectrode distance is shown in Fig. 4. As one can see, the temperature of the air flow had almost a linear dependence on the discharge current. Due to the fact that the power deposited into the discharge depended linearly on the discharge current (Fig. 3b), the linear dependence of temperature on the discharge current and, hence, on the power is obvious. It also follows from Fig. 4 that the interelectrode distance had no strong effect on the temperature of species leaving the conical electrode. At the same discharge current, the temperature change was within 1–2 °C when the interelectrode distance changed from 0 to 8 mm. As follows from the data in Fig. 3b, this is because the discharge current has a greater impact on the power than the interelectrode distance.

The maximum temperature of the air flow was recorded at the maximum discharge current of 500 μA and reached the value of 40 °C. At the discharge current of 200 μA, which was chosen as optimal for the NTP source, the temperature of species at the outlet of the conical electrode did not exceed 30 °C. Thus, the plasma generated by the discharge is safe in the view of thermal damage to living tissues.

As noted earlier, one of the advantageous features of the discharge in the needle-to-cone electrode configuration is that it blows out plasma species through the conical electrode without pumping any feed gas. The mass-transfer of active species from the discharge to the object being treated is of high importance, and therefore, its rate could be a factor determining the bactericidal properties of the discharge. In addition, the speed of ion wind depends on the number of ions generated by a discharge, and a higher number of ions may mean a higher number of active species. In particular, the dominant contribution to the induction of ion wind during a negative corona discharge in air is made by negative ions of the oxygen molecule. However, since superoxide is also an anion of the oxygen molecule but a radical^96^, one can expect a correlation between their concentrations. Note that superoxide is a ROS with strong bactericidal activity^59,97^. In this regard, one can assume that the speed of the ion wind should have a positive impact on the bactericidal properties of the discharge.

**Figure 5:**
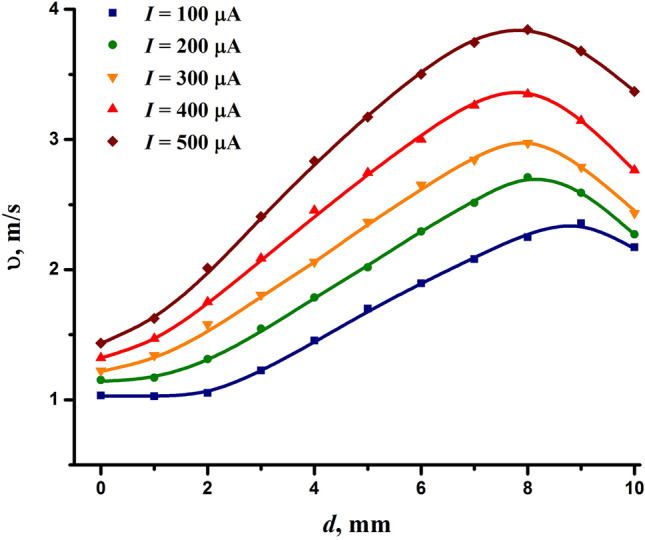
Speed of the ion wind induced by the discharge as a function of the interelectrode distance and the discharge current.

To reveal a possible correlation, we measured the speed of the ion wind at the outlet of the conical electrode. Figure 5 shows how the speed of the ion wind changes depending on the interelectrode distance and discharge current. As can be seen, the speed of the ion wind induced by the discharge can reach several meters per second. It decreased with reducing the interelectrode distance but still exceeded 1 m/s even at the shortest interelectrode gap of 0 mm.

The speed of the ion wind induced by a corona discharge can be described by the empirical expression^98–100^:

1 $$ \upsilon = k \cdot \sqrt {\frac{I}{\rho \cdot \mu }} , $$

where *I* is the discharge current, *ρ* is the gas density, *μ* is the ion mobility, and *k* is a factor that primarily depends on the geometry of the electrode system. Sigmond and Lagstad^101^ found that in the needle-to-plane electrode configuration, the factor $k = \sqrt{\frac{d}{A}}$, where *d* is the interelectrode distance and *A* is the cross-section area of the discharge. A theoretical justification for the expression (1) can be found in the work^102^. By studying the speed of the ion wind induced by a corona discharge in the needle-to-ring electrode configuration with interelectrode distances comparable to the diameter of the ring electrode, Li et al.^100^ noted that the cross-section area of the discharge is a complex function of the electrode parameters, and the factor *k* is difficult to obtain in analytical form.

As far as the discharge in the needle-to-cone electrode configuration is concerned, the derivation of the factor *k* is even more complex. However, the present research does not aim to address this issue. On the whole, the behavior of the ion wind speed (Fig. 5) was consistent with the expression (1). An increase in the discharge current led to an increase in the speed of the ion wind. As for the interelectrode distance, its increase also had a positive effect on the speed of the ion wind, but only until it reached approximately 8 mm, after which the speed began to decrease. The effect of the interelectrode distance on the speed of the ion wind is consistent with other works^100,103^ reporting that the speed reaches its maximum at the interelectrode distance, which is approximately equal to the radius of the ring electrode.

Comparing the data in Figs. 2 and 5, one can see no direct correlation between the ion wind speed and the bactericidal effect of the discharge. Although the bactericidal effect and the speed of the ion wind improved with increasing the discharge current, they were anticorrelated in terms of the interelectrode distance. Reducing the interelectrode distance, despite the decrease in the speed of the ion wind, had a strong positive impact on the bactericidal properties of the discharge. And vice versa, at the interelectrode distance corresponding to the maximum of the ion wind speed, the bactericidal effect was quite low. Thus, although the ion wind is important for the operation of the NTP source, its speed is not the decisive factor determining the bactericidal properties of the plasma source. Note that the lack of correlation may be due to various reasons and requires a more detailed study, which, however, is not the purpose of this research.

**Figure 6:**
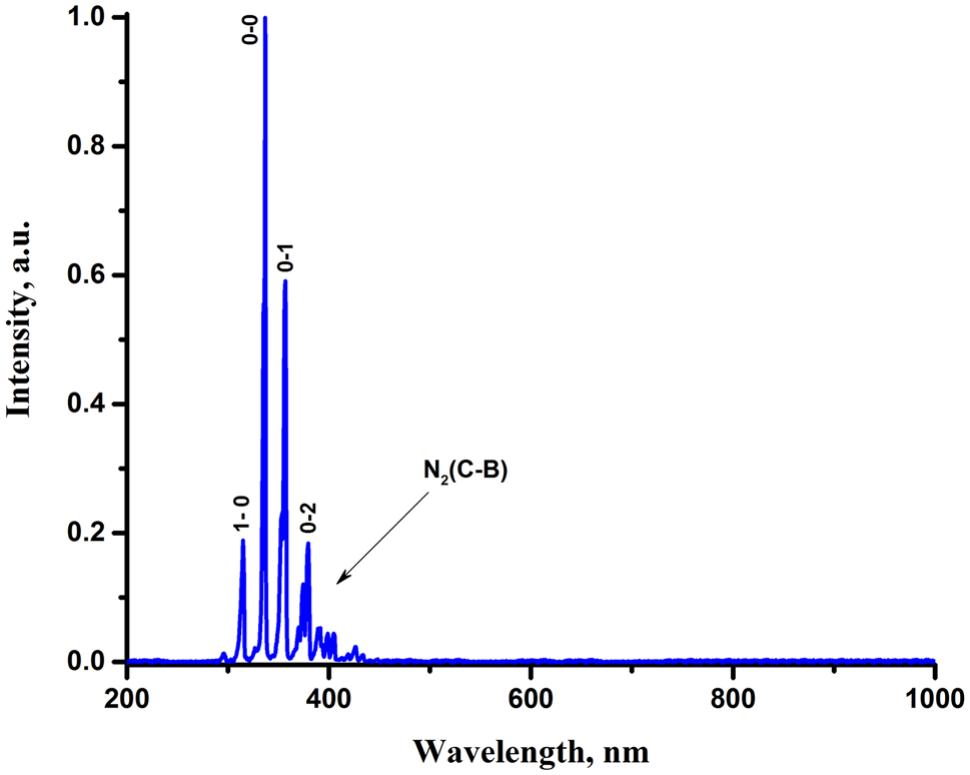
Emission spectrum of the generated plasma at the discharge current of 200 μA and the interelectrode distance of 0 mm.

Plasma diagnostics can help in identifying factors responsible for the bactericidal effect of the NTP. One of the most common methods of plasma diagnostics is the study of the composition of its radiation. We studied emission spectra of the generated plasma in the spectral range from 200 to 1000 nm with a change in the interelectrode distance and the discharge current. The recorded spectra corresponded to the region near the needle electrode since the radiation intensity in this region strongly prevails. Under the chosen optimal parameters of the discharge, i.e. at the discharge current of 200 μA and the interelectrode distance of 0 mm, the emission spectrum is virtually represented only by the second positive system of the nitrogen molecule (Fig. 6). The emission of the first negative system was also registered but its intensity was a few percent of the main peak in the spectrum. As for the emission of other plasma species, it was at the background level and was hardly detected in the spectrum.

The fact that the N_2_(C) nitrogen molecule is the main plasma species present in the emission spectrum indicates that the discharge efficiently produces the N_2_(A) metastable nitrogen molecules, which is consistent with other works^64,104–106^. Being a long-lived state with a radiative lifetime of 2 s^107^, the N_2_(A) metastable nitrogen molecules virtually do not spontaneously de-excite (due to the forbiddance of the optical transition from the metastable state to the ground state). Although the metastable state cannot decay on its own, it can be quenched upon collision, transferring its excitation energy to a colliding species.

It is well known that the N_2_(A) metastable state plays an important role in the kinetics of an atmospheric plasma^64,108–110^. Having the excitation energy of 6.17 eV^107^, the N_2_(A) metastable nitrogen molecules can trigger various plasma-chemical processes and, in particular, form such biomedically active species as O, O_3_, NO, NO_2_, OH, and H_2_O_2_. For instance, the energy of the N_2_(A) metastable state is sufficient to cause the dissociation of molecular oxygen (5.17 eV), which results in the formation of highly reactive atomic oxygen^62,64,108^:

2 $$ {\text{N}}_{{2}} {\text{(A)}} + {\text{O}}_{{2}} \to {\text{N}}_{{2}} {\text{(X)}} + {\text{O}} + {\text{O}}{.} $$

In addition, the resulting atomic oxygen can further react with molecular oxygen and form highly oxidizing ozone through the association reaction^62,110^:

3 $$ {\text{O}} + {\text{O}}_{{2}} + {\text{M}} \to {\text{O}}_{{3}} + {\text{M}}{.} $$

Furthermore, metastable molecular nitrogen, reacting with atomic oxygen, can form nitric oxide^62,64,108,110^:

4 $$ {\text{N}}_{{2}} {\text{(A)}} + {\text{O}} \to {\text{NO}} + {\text{N(}}{}^{{2}}{\text{D)}}{.} $$

Nitrogen dioxide is usually formed by the interaction of nitric oxide with ozone^62,64^:

5 $$ {\text{NO}} + {\text{O}}_{{3}} \to {\text{O}}_{{2}} + {\text{NO}}_{{2}} , $$

and as a result of the association reaction of nitric oxide with atomic oxygen^62,110^:

6 $$ {\text{NO}} + {\text{O}} + {\text{M}} \to {\text{NO}}_{{2}} + {\text{M}}{.} $$

In the reaction with molecular oxygen, metastable nitrogen molecules can form nitrous oxide:

7 $$ {\text{N}}_{{2}} {\text{(A)}} + {\text{O}}_{{2}} \to {\text{N}}_{{2}} {\text{O}} + {\text{O}}{.} $$

As reported in the works^64,111^, the reaction (7) is one of the main mechanisms for the formation of nitrous oxide at a high content of metastable nitrogen molecules.

The N_2_(A) metastable molecular nitrogen can also cause the dissociation of a water molecule (5.15 eV) and form a highly reactive hydroxyl radical^62,112,113^:

8 $$ {\text{N}}_{{2}} {\text{(A)}} + {\text{H}}_{{2}} {\text{O}} \to {\text{N}}_{{2}} {\text{(X)}} + {\text{OH}} + {\text{H}}{.} $$

Finally, the resulting hydroxyl radicals can further form hydrogen peroxide through the association reaction as follows^62,110^:

9 $$ {\text{OH}} + {\text{OH}} + {\text{M}} \to {\text{H}}_{{2}} {\text{O}}_{2} + {\text{M}}{.} $$

Also of note is the fact that the above reactions are post-discharge reactions, i.e., they can proceed after the discharge or outside the discharge region^62,64,109,114,115^, which is of great importance. Due to the relatively large distance (~ 1 cm) from the discharge to the treatment object, only long-lived active species can reach the object. However, through long-lived metastable molecular nitrogen entrained by the ion wind, both long-lived and short-lived reactive species can be formed right at the object being processed.

As is clear, a discharge in the air can generate various active species, and the pathways for their formation can be very diverse. It is worth mentioning that the active species are closely related to each other, sometimes even converting from one into another, and their content in a plasma is determined by the competition of many plasma-chemical processes^58,62–64^.

To date, the question of the role of different active species is still acute and the controversy is still ongoing on this issue. Some studies (e.g.^46^) claim that ROS play a key role in bacteria inactivation, and others (e.g.^116–120^) state that RNS are extremely important, especially peroxynitrite, which is believed to be the species most responsible for the bactericidal effect in plasma-activated water^58,121–123^. A lot of works have been devoted to the study of the bactericidal properties of ozone. Although most of them indicate a strong inactivating effect of ozone (e.g.^124–126^), one can come across papers stating that the contribution of ozone to the bactericidal effect is negligible (e.g.^116,118,120,127^). Furthermore, Dobrynin et al.^128^ came to the conclusion that neither UV radiation, ozone, hydrogen peroxide, nor other neutral reactive species are responsible for the bactericidal effect of the corona discharge. Such contradictory statements seem to be due to the fact that NTP sources developed in different research laboratories are unique and have their own specific properties and characteristics. In this regard, the diagnostics of reactive species generated by the discharge system used in our research is of fundamental importance.

**Figure 7:**
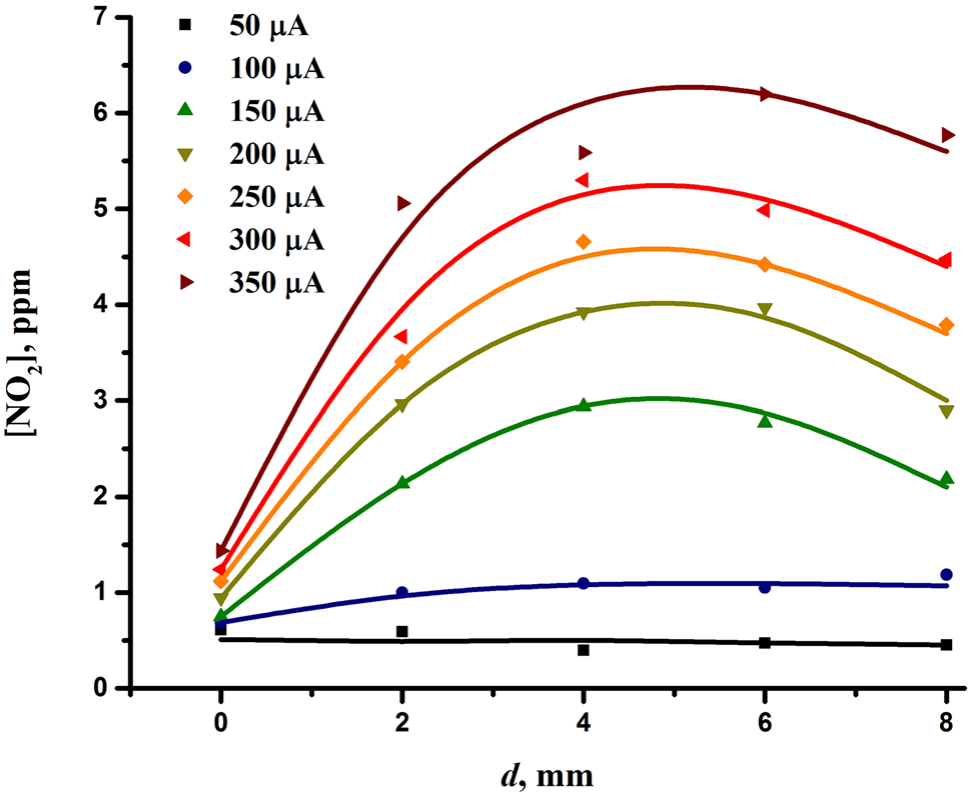
Concentration of nitrogen dioxide generated by the discharge as a function of the interelectrode distance and the discharge current.

The results of measuring the concentration of nitrogen dioxide generated by the discharge are shown in Fig. 7. The graph displays the effect of the discharge current and the interelectrode distance on the production of NO_2_. As can be seen, the concentration of nitrogen dioxide was on the order of several parts per million. In particular, under the chosen optimal discharge conditions, i.e. at the discharge current of 200 μA and the interelectrode distance of 0 mm, the concentration of nitrogen dioxide was only about 1 ppm. An increase in the discharge current had a beneficial effect on the production of nitrogen dioxide. However, the measurements indicate a quite uneven influence of the discharge current on the NO_2_ yield. Although the generation of nitrogen oxides is usually proportional to the power deposited into the discharge (e.g.^61,129^), which in our case has virtually a linear dependence on the discharge current (Fig. 3b), Fig. 7 clearly shows the difference in the amount of NO_2_ formed at the discharge currents below and above 150 μA. Taking into account the volt-ampere characteristics of the discharge (Fig. 3a), one can conclude that this difference should be due to the change in the discharge mode.

As for the influence of the interelectrode distance, its increase also had a positive impact on the production of nitrogen dioxide but only up to distances of approximately 4–5 mm. A further increase in the interelectrode spacing led to a decrease in the concentration of nitrogen dioxide, despite the increase in the power delivered into the discharge with the interelectrode distance (Fig. 3b). However, for the formation of nitrogen oxides, the specific power is of importance, which depends on the active volume of the corona discharge. The volume of the ionization region of a corona discharge can increase with the discharge current^130^ and voltage^74^. An increase in the interelectrode distance is accompanied by an increase in the discharge voltage (Fig. 3a), which can increase the active volume of the corona discharge, leading to a decrease in the specific power deposited into the plasma.

As far as the concentrations of higher oxides of nitrogen (NO_x_) and nitric oxide (NO) are concerned, they were not detected over the entire range of experimental conditions. Note that the detection limit of the NO_x_ analyzer used is 50 ppb. The low generation of nitric oxide is quite obvious as it requires relatively high gas temperatures and a higher degree of nitrogen dissociation, which can be achieved with higher discharge currents, in particular in the transient spark discharge mode^61,131^.

Comparing the behavior of the bactericidal effect of the discharge (Fig. 2) and the concentration of nitrogen dioxide (Fig. 7) depending on the discharge current and interelectrode distance, one can see that they have an inverse dependence on the interelectrode distance. In particular, at the interelectrode distance of 0 mm, at which the bactericidal effect is at its highest, the concentration of nitrogen dioxide is minimal. Thus, it follows that nitrogen oxides do not play a key role in the bactericidal effect of the discharge.

**Figure 8:**
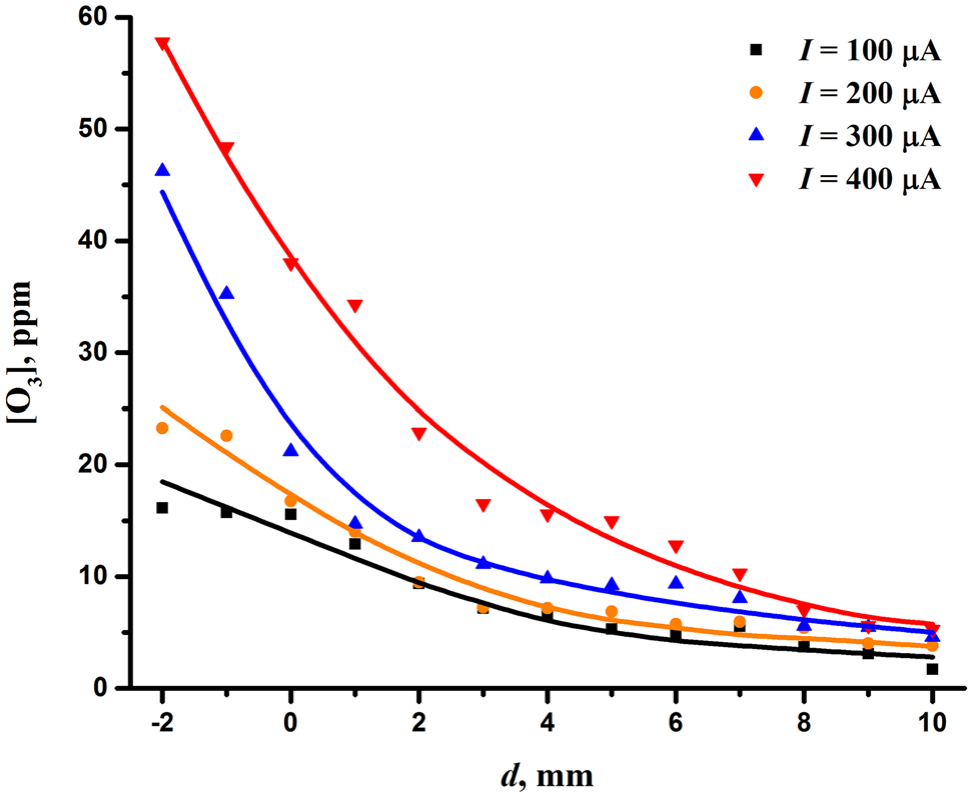
Concentration of ozone produced by the discharge as a function of the interelectrode distance and the discharge current.

Figure 8 shows the formation of ozone depending on the interelectrode distance and discharge current. As can be seen, the ozone concentration was on the order of several tens of parts per million and at the chosen optimal discharge conditions, it was slightly less than 20 ppm. Thus, ozone prevails over nitrogen oxides. In fact, the predominance of ozone at low production of nitric oxide is obvious^61,116,118,131^. As is known (e.g.^61,132^), ozone is effectively decomposed by nitric oxide in the reaction (5) and under the influence of high temperatures necessary for the productive generation of nitric oxide. Depending on discharge conditions, this usually leads to a predominance of either ozone or nitric oxide in an air plasma.

Analysis of the dependence in Fig. 8 indicates that the ozone yield responds positively to an increase in the discharge current, while an increase in the interelectrode distance, on the contrary, leads to its decrease. Thus, one can see a clear correlation in the behavior of the bactericidal effect (Fig. 2) and ozone generation depending on both the discharge current and the interelectrode distance. This fact indicates that ozone may play a key role in the bactericidal effect of the discharge.

Of particular interest is the role of hydrogen peroxide in the bactericidal effect of the discharge. Hydrogen peroxide is a stable and long-lived ROS with well-known antibacterial properties. To detect hydrogen peroxide as well as nitrite and nitrate, we employed test strips used to diagnose plasma-activated water. It turned out that when Petri dishes with agar were exposed to the NTP, humidity increased high enough to activate the test strips in the gas phase. However, because test strips are used to evaluate the content of chemicals in liquids and give concentrations in milligrams per liter, the values obtained cannot be directly related to airborne concentrations. Therefore, the concentrations of hydrogen peroxide as well as nitrite and nitrate presented in the following graphs are given in relative units. Note that the values on the axes correspond to those in milligrams per liter given by the test strips, so they can be compared.

**Figure 9:**
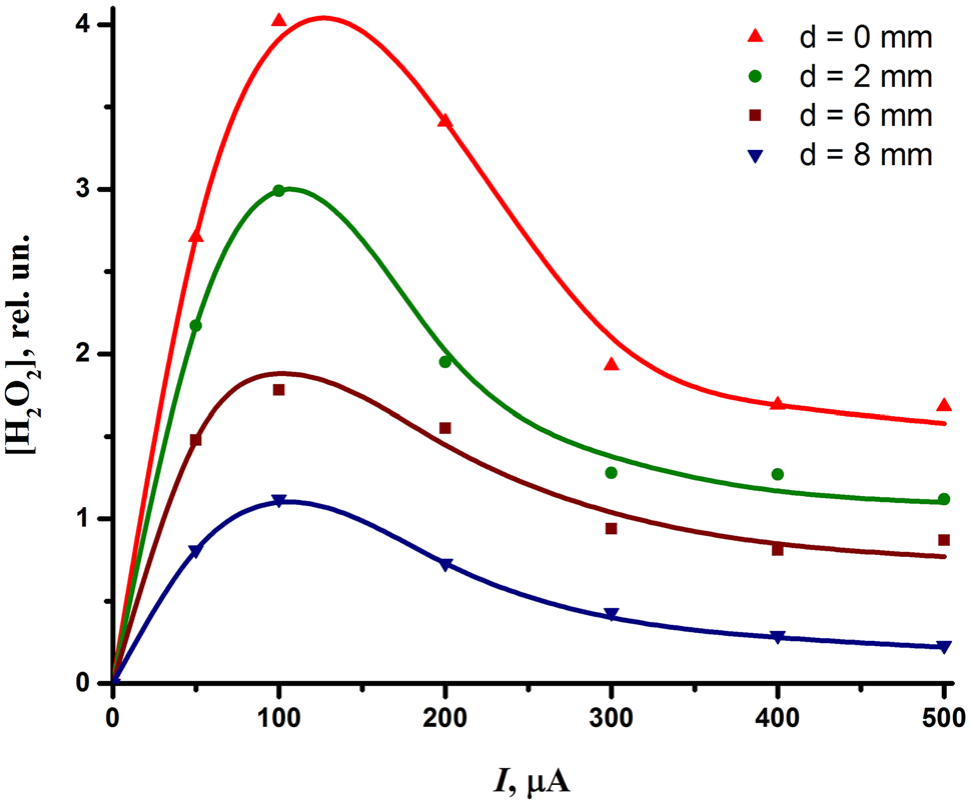
Influence of the discharge current and interelectrode distance on the production of hydrogen peroxide.

Figure 9 illustrates the formation of hydrogen peroxide depending on the discharge current and interelectrode distance. As follows from the graph, the formation of hydrogen peroxide had an inverse dependence on the interelectrode distance just like the bactericidal effect of the discharge (Fig. 2). However, the discharge current had a different impact on the formation of hydrogen peroxide and the bactericidal properties of the discharge. An increase in the discharge current above 100 μA caused the yield of hydrogen peroxide to decrease, while in terms of the bactericidal properties of the discharge, the current had only a positive effect (Fig. 2). Thus, hydrogen peroxide can be excluded from the reactive species directly responsible for the bactericidal effect of the discharge, and, therefore, its more precise measurement is not required. Note that this finding does not mean that hydrogen peroxide can be neglected in other plasma sources, since most of them are unique and produce plasmas with different compositions of reactive species.

**Figure 10:**
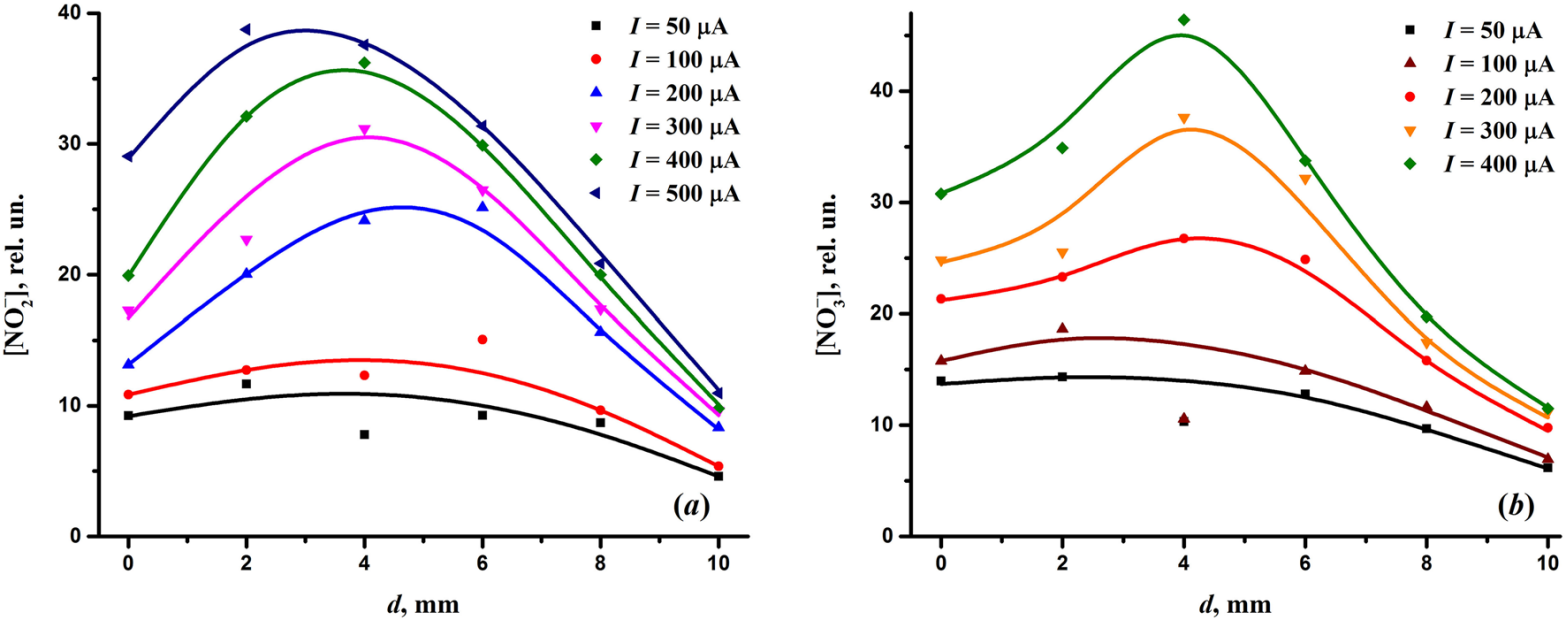
Influence of the discharge current and interelectrode distance on the formation of nitrites (**a**) and nitrates (**b**).

When studying plasma-activated water, attention is also paid to such long-lived reactive species as nitrites (${\text{NO}}_{{2}}^{ - }$) and nitrates (${\text{NO}}_{{3}}^{ - }$), which affect its bactericidal properties. We studied their formation by the discharge in the gas phase. The effect of the discharge current and the interelectrode distance on the formation of nitrites and nitrates is shown in Fig. 10. As can be seen, the content of nitrites and nitrates was close. Moreover, the formation of nitrites and nitrates was basically similar in nature with respect to the influence of the discharge current and the interelectrode distance. As for the discharge current, it benefits the production of both nitrites and nitrates. However, as in the case of nitrogen dioxide, the effect of the discharge current on the formation of nitrites and nitrates was rather uneven. It is clearly seen (Fig. 10) the difference in the formation of ${\text{NO}}_{{2}}^{ - }$ and ${\text{NO}}_{{3}}^{ - }$ at the discharge currents below and above 150 μA, which is associated with the change in the discharge mode (see Fig. 3a).

As far as the interelectrode distance is concerned, its increase also had a positive effect on the formation of both nitrites and nitrates but only up to distances of approximately 4 mm. Its further increase caused the formation of ${\text{NO}}_{{2}}^{ - }$ and ${\text{NO}}_{{3}}^{ - }$ to decrease, which was associated with the decrease in the generation of nitrogen dioxide (Fig. 7). It is necessary to note the similarity in the formation of nitrites, nitrates and nitrogen dioxide depending on both the discharge current and the interelectrode distance. The correlation between nitrites, nitrates and nitrogen dioxide indicates their close relationship resulting from the reaction^61,123^:

10 $$ {\text{NO}}_{{2}} + {\text{NO}}_{{2}} + {\text{H}}_{{2}} {\text{O}} \to {\text{NO}}_{{2}}^{ - } + {\text{NO}}_{{3}}^{ - } + 2{\text{H}}^{ + } . $$

Although the formation of nitrites and nitrates correlated with the bactericidal effect in terms of the influence of the discharge current, they responded differently to the change in the interelectrode distance. The lack of a total correlation between the bactericidal effect and the formation of nitrites/nitrates indicates that they are not the species directly responsible for the bactericidal properties of the discharge.

The presence of ${\text{NO}}_{{2}}^{ - }$ and ${\text{NO}}_{{3}}^{ - }$ is usually associated with the presence of HNO_2_ and HNO_3_ acids. An increase in the content of acids, hydrogen peroxide, and other reactive species in plasma-activated water affects its pH value. We also tried to measure the pH of the gaseous medium using test strips, but the wet gas was unable to activate the pH test strips used.

Unfortunately, we are not able to measure all known reactive species since almost each of them requires special equipment. It is generally accepted that nitrogen oxides, nitrites, nitrates, peroxynitrites and peroxynitrous acid are the main reactive species among RNS, which can play a significant role in the inhibition of microorganisms^133–135^. It has been shown that nitrogen oxides, nitrites and nitrates are not the species directly responsible for the bactericidal effect of the discharge. As for peroxynitrite (O=NOO^–^), it can be formed in the reaction of superoxide with nitric oxide^60,123,133,136^:

11 $$ {\text{O}}_{{2}}^{ - } + {\text{NO}} \to {\text{O}} = {\text{NOO}}^{ - } , $$

and through the reaction of hydroxyl radicals with nitrogen dioxide^123^:

12 $$ {\text{OH}} + {\text{NO}}_{{2}} \to {\text{O}} = {\text{NOO}}^{ - } + {\text{H}}^{ + } . $$

Due to the low content of nitric oxides, the formation of peroxynitrite by the reaction (11) can be excluded. The productivity of the reaction (12) is also questionable. On the one hand, hydroxyl radical is a short-lived species with the lifetime of approximately 200 μs in the gas phase and on the order of a few nanoseconds in the liquid phase^136,137^. On the other hand, the production of nitrogen dioxide, which is the second reagent in the reaction (12), did not totally correlate with the bactericidal effect of the discharge. Nevertheless, the formation of peroxynitrite may take place, but it is unlikely that peroxynitrite is the main species responsible for the bactericidal effect of the discharge.

Peroxynitrous acid (O=NOOH) can be formed also in the reaction of hydroxyl radicals with nitrogen dioxide^61,123,133^:

13 $$ {\text{OH}} + {\text{NO}}_{{2}} \to {\text{O}} = {\text{NOOH,}} $$

and through the reaction of nitrites with hydrogen peroxide and hydrogen ions^61,121,123,136,138^

14 $$ {\text{NO}}_{{2}}^{ - } + {\text{H}}_{2} {\text{O}}_{{2}} + {\text{H}}^{ + } \to {\text{O}} = {\text{NOOH}} + {\text{H}}_{2} {\text{O}}{.} $$

Due to the same reagents, the reaction (13) should have similar productivity as the reaction (12). The reaction (14) is also questionable because it requires three reagents, none of which is in abundance. In addition, the formation of nitrites and hydrogen peroxide did not totally correlate with the bactericidal effect, indicating that peroxynitrous acid is unlikely to play a key role in the bactericidal properties of the discharge. Thus, RNS are not the main species responsible for the bactericidal effect.

Some studies point to UV radiation of the discharge as a potent bactericidal agent. In our case, UV radiation can be excluded from the decisive factors, since 97% of the Petri dish area is beyond the reach of direct rays form the discharge.

As far as ROS are concerned, an important role in the inactivation of microorganisms can play atomic oxygen, singlet oxygen, superoxides, ozone, hydroxyl radicals, and hydrogen peroxide^133–135^. Among the measured ROS, which were ozone and hydrogen peroxide, only ozone correlated well with the bactericidal properties of the discharge, indicating its role as the primary mediator. This finding is consistent with other studies (e.g.^139^) reporting a correlation of the bactericidal effect with the ozone concentration in the corona discharge. However, due to the lack of data on the other ROS, their role cannot be completely neglected, despite the strong correlation found between the content of ozone and the bactericidal effect. In particular, one can expect a correlation between ozone and atomic oxygen, which is a necessary reagent for ozone formation in the association reaction (3). Therefore, the bactericidal effect of the discharge should generally be attributed to ROS.

### Design of the plasma source

**Figure 11:**
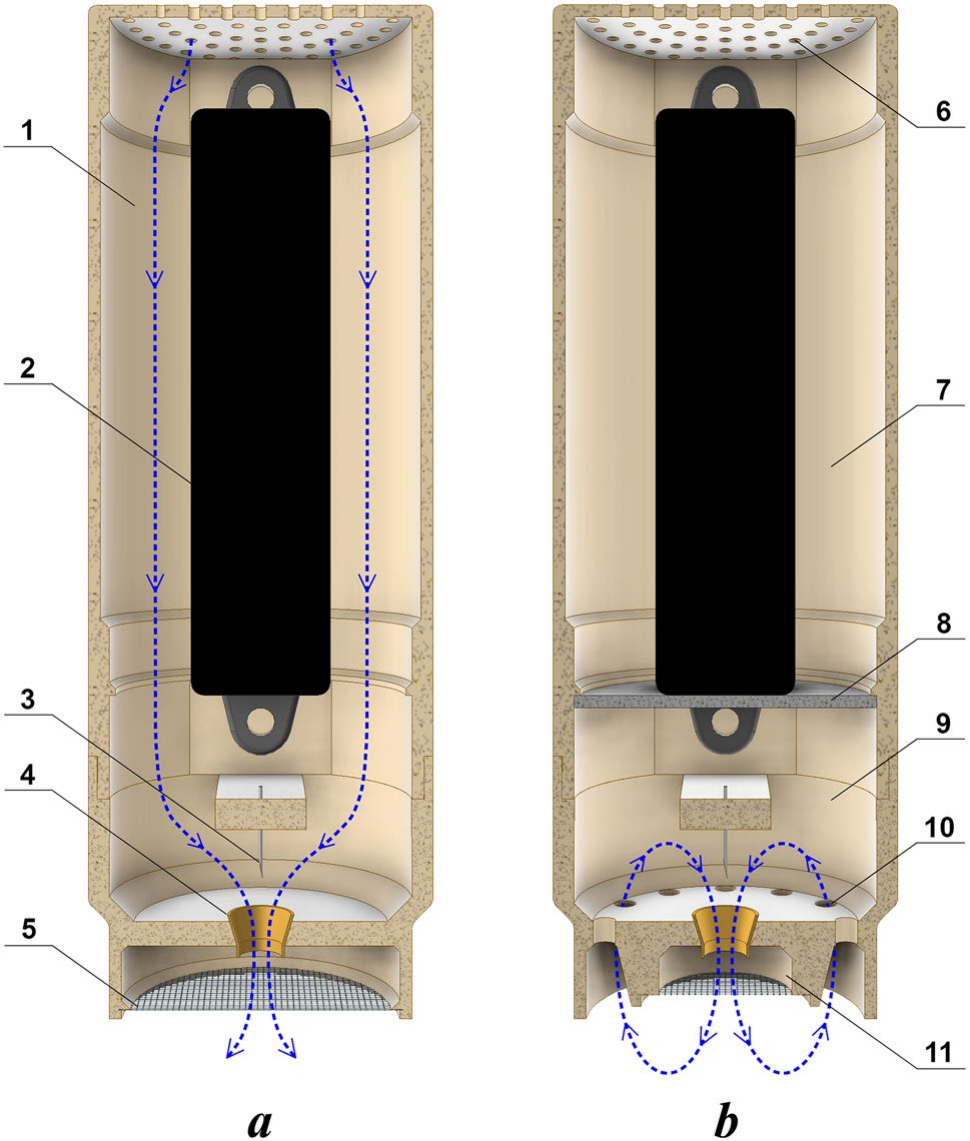
Designs of the NTP source: (**a**) initial non-optimized design and (**b**) final design. 1—case of the plasma source, 2—high-voltage power supply, 3—needle electrode, 4—conical electrode, 5—mesh, 6—holes for convection cooling of the high-voltage power supply, 7—power supply section, 8—partition wall, 9—discharge section, 10—holes for return flow of active species, 11—plasma source outlet with a mesh.

The initial design of the NTP source was just the discharge and a high-voltage power supply enclosed in a compact cylindrical case 3D-printed of PETG plastic. The design is shown schematically in Fig. 11a. The mesh under the conical electrode served, on the one hand, to protect against touching the high-voltage electrode and, on the other hand, to distribute active species blown out by the discharge over a larger treatment area. The effect of a mesh on increasing the area of inhibition was reported in the work^91^.

Despite such advantages of the NTP source as simplicity and ease of manufacture, its initial design had shortcomings that reduced the efficiency of the plasma source. In particular, one of the biggest shortcomings was that it was difficult for the discharge to blow active species through the conical electrode when the plasma source was connected to a closed volume. Obviously, the net flow through the conical electrode in this case should be zero. To eliminate this problem, we have changed the design of the plasma source, allowing the flow of active species to circulate freely. The modified design of the NTP source is shown schematically in Fig. 11b.

In principle, it is sufficient to have holes in the bottom wall of the plasma source to allow free circulation of active species. However, we found that further changes are needed. In particular, the mesh at the outlet of the plasma source is a kind of barrier that returns some part of active species to the plasma source without leaving the mesh. In order to eliminate this problem, the mesh was placed exclusively at the outlet of the conical electrode, giving no way for active species to get to the backflow holes other than through the mesh (Fig. 11b).

In addition, we introduced a partition wall inside the plasma source and found that it also plays an important role. On the one hand, this prevents leakage of active species through the holes in the upper wall of the NTP source, which are designed mainly for convection cooling of the high-voltage power supply. On the other hand, the partition wall reduces the volume of active species inside the plasma source and, therefore, increases their concentration.

The changes made to the design of the NTP source have been tested for their effect on the bactericidal properties of the plasma source. We investigated the inactivating effect of the NTP source on *E. coli* and found that each change made to the design of the plasma source noticeably enhanced its bactericidal ability.

### Testing of the NTP source

The developed NTP source has been optimized for maximum bactericidal action using *E. coli*. However, the plasma source is intended to be used for a wide range of biomedical applications, so it must be effective against other pathogens as well. To explore the bactericidal ability of the developed NTP source, we tested its inactivating effect on different types of microorganisms, in particular, microfungi, yeast, gram-positive and gram-negative bacteria. We used *P. aeruginosa* as a representative of gram-negative bacteria, *S. aureus* as a representative of gram-positive bacteria, *C. albicans* as a representative of yeasts, and *T. interdigitale* as a representative of microfungi. We also used the extremophilic bacterium *D. radiodurans*, which is known to be one of the most resistant living organisms to many DNA-damaging agents, including ultraviolet and even ionizing radiation^140–143^.

**Figure 12:**
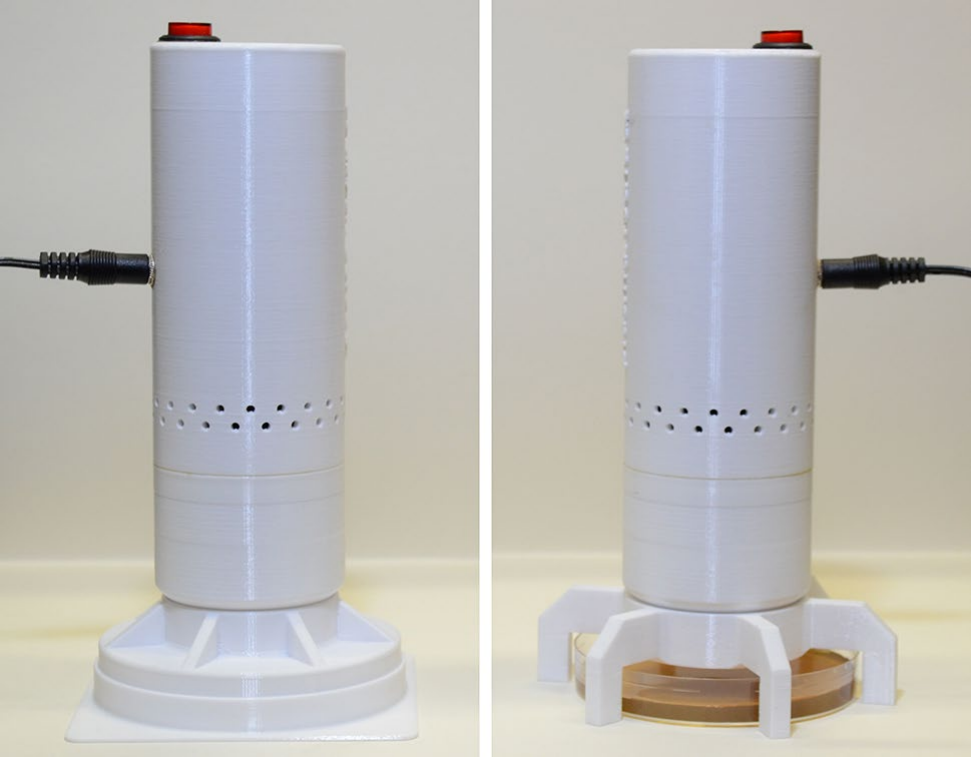
The NTP source used in the closed-volume mode (on the left) and in the open-air mode (on the right).

**Figure 13:**
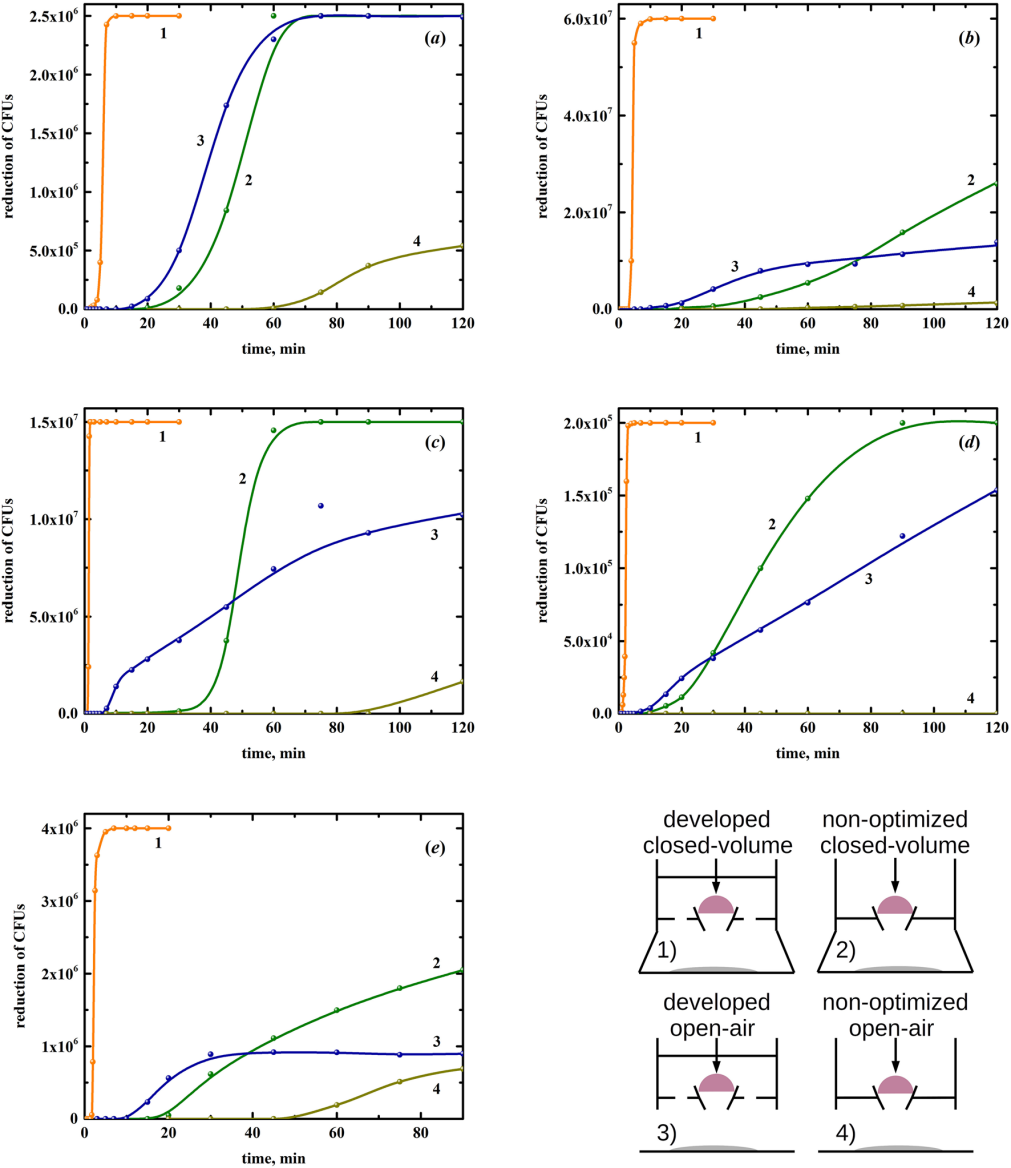
Decrease in CFUs of *P. aeruginosa* (**a**), *S. aureus* (**b**), *C. albicans* (**c**), *T. interdigitale* (**d**), and *D. radiodurans* (**e**) depending on the time of exposure to the developed NTP source in the closed-volume mode (1), non-optimized NTP source in the closed-volume mode (2), developed NTP source in the open-air mode (3), and to the non-optimized NTP source in the open-air mode (4). In the right bottom corner, a schematic drawing of the tested configurations is given.

On the other hand, the NTP source, in the view of practical application, may be used in two ways. The most preferred way is when an object is enclosed in the treatment volume of the plasma source. Unfortunately, some objects can be treated only in the open air because, due to geometric or other restrictions, they do not allow the formation of a closed treatment volume. In this regard, the bactericidal ability of the plasma source in both of these cases is of great importance. Therefore, we tested the inactivating effect of the NTP source on the above microorganisms both in the closed-volume and open-air modes. Both the modes are implemented by mounting 3D-printed applicators onto the NTP source (Fig. 12). In addition, it was of interest to compare the bactericidal ability of the developed NTP source with the original non-optimized NTP source. Recall that the original non-optimized NTP source has the interelectrode distance of approximately 3.3 mm, the discharge current of 150 μA, and the design shown in Fig. 11a. The test results are presented in Fig. 13.

Comparing the developed and original non-optimized NTP source, one can see a significant difference in their bactericidal ability (Fig. 13). The developed NTP source was superior to the original non-optimized NTP source both in the closed-volume and open-air modes. Roughly speaking, the developed NTP source in the open-air mode and the original non-optimized NTP source in the closed-volume mode provided a comparable bactericidal effect despite a huge difference between inactivation capability in the closed-volume and open-air modes for the same NTP source (Fig. 13).

The open-air mode has a more pronounced local bactericidal effect in the center of a Petri dish since the plasma species are blown out of the conical electrode in a narrow flow. In a case of a large number of CFUs inoculated into a Petri dish or the use of more resistant microorganisms, it is difficult for the NTP source in the open-air mode to eradicate the microorganisms at the edges of a Petri dish since the diameter of the Petri dish is much larger than the effective diameter of the plasma species flow and only a small number of active species reach the periphery of the Petri dish. That is why the developed NTP source in the open-air mode did not completely eradicate microorganisms in some cases (curve 3 in Fig. 13). This effect was particularly noticeable when testing the NTP source in the open-air mode on *D. radiodurans* (Fig. 13e). After 30 min of exposure, the decrease in CFUs almost stopped. *D. radiodurans* was inactivated in the center of the Petri dishes but survived at the edges, indicating the unevenness of the bactericidal effect over the Petri dish area.

Unlike the open-air mode, the NTP source in the closed-volume mode has a considerably higher inactivation capability. By preventing active species from dissipating into the environment, the closed volume promotes their accumulation, which has a decisive impact on the bactericidal ability of the NTP source. Thus, using the NTP source in the open-air mode should be avoided if possible. In addition, the closed-volume mode has a safety advantage because it prevents the release of ozone and nitrogen oxides into the environment, which are harmful when inhaled.

Thus, the test results showed that, despite the rather low power of 1.4 W deposited into the discharge, the developed NTP source quite well inactivates, in addition to *E. coli*, other microorganisms, in particular, microfungi, yeasts, gram-positive and gram-negative bacteria. In the case of the closed-volume mode, the developed NTP source eradicated the tested microorganisms within one to several minutes, depending on a microorganism and its number. The number of CFUs inoculated into a Petri dish was as follows: 2.5 × 10^6^ CFUs of *P. aeruginosa*, 6 × 10^7^ CFUs of *S. aureus*, 1.5 × 10^7^ CFUs of *C. albicans*, 2 × 10^5^ CFUs of *T. interdigitale*, and 4 × 10^6^ CFUs of *D. radiodurans*.

It is worth mentioning that although germicidal lamps are ineffective against the extremophilic bacterium *D. radiodurans*^142^, the developed plasma source has been shown to effectively inactivate it. Also particularly significant is that the NTP source inhibits micromycetes represented here by *T. interdigitale*, which was the most resistant microorganism among those tested. Due to the protective outer coating, micromycetes are highly resistant to various factors, which makes it difficult to completely eradicate them by conventional methods.

## Conclusions

The paper details the development and characterization of a low-cost handheld source of a cold air plasma intended for biomedical applications. The plasma source is based on a DC discharge in the needle-to-cone electrode configuration and is an extremely simple device, essentially consisting of two electrodes and a power supply. The plasma source can be easily reproduced by anyone with minimal equipment and experience in working with high voltage^94^.

The discharge underlying the operation of the NTP source has been optimized for the best bactericidal effect on *E. coli*. The results showed that the bactericidal effect of the discharge significantly enhanced with increasing the discharge current and reducing the interelectrode distance. The parameters of the discharge for the developed NTP source are 0 mm for the interelectrode distance and 200 μA for the discharge current.

To characterize the discharge, we studied its electrical characteristics, emission spectra, the speed of the induced ion wind, temperature of species at the outlet of the conical electrode, formation of ozone, nitrogen oxides, hydrogen peroxide, nitrite, and nitrate, depending on both the discharge current and interelectrode distance. It was shown that RNS and UV radiation are not directly responsible for the bactericidal effect of the discharge. Among the reactive species studied, ozone was found to correlate well with the bactericidal effect, indicating that it is the key mediator in the inactivating effect of the plasma source. However, due to the lack of data on the other ROS, their role cannot be completely neglected, despite the strong correlation found between the content of ozone and the bactericidal effect. Therefore, the bactericidal effect of the discharge should generally be attributed to ROS. Nevertheless, ozone can be used as an indicator of the bactericidal effect if further optimization or modification of the plasma source is considered.

Attention was also paid to the design of the plasma source. By identifying and subsequently eliminating factors that limited the operation of the NTP source, an additional increase in its bactericidal ability was achieved. The NTP source can be used both in the open-air and closed-volume modes. The latter mode is implemented by mounting a 3D-printed applicator on the NTP source and provides the best bactericidal effect. In addition, the closed-volume mode is also preferred in the view of safety as it prevents the release of ozone and nitrogen oxides into the environment, which are harmful when inhaled.

The developed NTP source was tested on various types of microorganisms to verify its bactericidal ability. The testing showed that despite the rather low electrical power of only 1.4 W supplied to the discharge, the plasma source inactivates a wide range of pathogens, including microfungi, yeasts, gram-positive and gram-negative bacteria. Particularly significant is that the plasma source inhibits the extremophilic bacterium *D. radiodurans*, which is one of the most resistant living organisms to many DNA-damaging agents, including ultraviolet and even ionizing radiation. Note that the developed NTP source has great potential for many other applications, although customized discharge optimization could be beneficial to attain the best effect in a particular application.

Thus, in addition to developing a portable and affordable NTP source that can be reproduced by anyone, the paper reveals the bactericidal effect of a corona discharge in air and the formation of long-lived ROS and RNS by the discharge, depending on both the interelectrode distance and the discharge current. To the best of our knowledge, this is the first such comprehensive study of the bactericidal properties of a corona discharge in air.

## Data Availability

The raw data underlying the findings presented in this research are available from the corresponding author upon request. Technical information as well as a detailed guide to creating the NTP source are available at the following URL: https://ufmt.vscht.cz/pns.

## References

[1] Scholtz V (2023). Non-thermal plasma disinfecting procedure is harmless to delicate items of everyday use. Sci. Rep..

[2] Chu PK, Lu X (2013). Low Temperature Plasma Technology: Methods and Applications.

[3] Chen Z, Wirz RE (2021). Cold Atmospheric Plasma (CAP) Technology and Applications.

[4] Okubo M (2023). Nonthermal Plasma Surface Modification of Materials.

[5] Güçeri S, Fridman A, Gibson K, Haas Ch (2008). Plasma Assisted Decontamination of Biological and Chemical Agents.

[6] Machala Z, Hensel K, Akishev Y (2012). Plasma for Bio-decontamination, Medicine and Food Security.

[7] Misra NN, Schlüter O, Cullen PJ (2016). Cold Plasma in Food and Agriculture: Fundamentals and Applications.

[8] Laroussi M (2021). Low-temperature plasma for biology, hygiene, and medicine: Perspective and roadmap. IEEE Trans. Radiat. Plasma Med. Sci..

[9] Ekezie FGC, Sun DW, Cheng JH (2017). A review on recent advances in cold plasma technology for the food industry: Current applications and future trends. Trends Food Sci. Technol..

[10] Zille A, Oliveira FR, Souto AP (2015). Plasma treatment in textile industry. Plasma Process. Polym..

[11] Chang JS (2001). Recent development of plasma pollution control technology: A critical review. Sci. Technol. Adv. Mater..

[12] Chen Z (2022). Cold atmospheric plasma delivery for biomedical applications. Mater. Today.

[13] von Woedtke T, Laroussi M, Gherardi M (2022). Foundations of plasmas for medical applications. Plasma Sources Sci. Technol..

[14] Weltmann KD, von Woedtke T (2016). Plasma medicine—Current state of research and medical application. Plasma Phys. Control. Fus..

[15] Fridman G (2008). Applied plasma medicine. Plasma Process. Polym..

[16] Kong MG (2009). Plasma medicine: An introductory review. New J. Phys..

[17] Duarte S, Panariello BH (2020). Comprehensive biomedical applications of low temperature plasmas. Arch. Biochem. Biophys..

[18] Shintani H, Sakudo A, Burke P, McDonnell G (2010). Gas plasma sterilization of microorganisms and mechanisms of action (review). Exp. Ther. Med..

[19] Shintani H (2007). Inactivation of microorganisms and endotoxins by low temperature nitrogen gas plasma exposure. Biocontrol Sci..

[20] Kolb JF (2008). Cold atmospheric pressure air plasma jet for medical applications. Appl. Phys. Lett..

[21] Maho T (2021). Anti-bacterial action of plasma multi-jets in the context of chronic wound healing. Appl. Sci..

[22] Tipa RS, Kroesen GM (2011). Plasma-stimulated wound healing. IEEE Trans. Plasma Sci..

[23] Shimizu T (2020). Wound treatment by low-temperature atmospheric plasmas and issues in plasma engineering for plasma medicine. Jpn. J. Appl. Phys..

[24] Nastuta AV, Topala I, Grigoras C, Pohoata V, Popa G (2011). Stimulation of wound healing by helium atmospheric pressure plasma treatment. J. Phys. D Appl. Phys..

[25] Haertel B, von Woedtke T, Weltmann KD, Lindequist U (2014). Non-thermal atmospheric-pressure plasma possible application in wound healing. Biomol. Ther..

[26] Lloyd G (2010). Gas plasma: Medical uses and developments in wound care. Plasma Process. Polym..

[27] Huang J (2011). Dielectric barrier discharge plasma in Ar/O_2_ promoting apoptosis behavior in A549 cancer cells. Appl. Phys. Lett..

[28] Semmler ML (2020). Molecular mechanisms of the efficacy of cold atmospheric pressure plasma (CAP) in cancer treatment. Cancers.

[29] Keidar M (2015). Plasma for cancer treatment. Plasma Sources Sci. Technol..

[30] Babington P (2015). Use of cold atmospheric plasma in the treatment of cancer. Biointerphases.

[31] Ratovitski EA (2014). Anti-cancer therapies of 21st century: Novel approach to treat human cancers using cold atmospheric plasma. Plasma Process. Polym..

[32] Friedman PC (2020). From precancers to skin rejuvenation—A review of the wide spectrum of current applications and future possibilities for plasma dermatology. Plasma Med..

[33] Heinlin J (2011). Plasma applications in medicine with a special focus on dermatology. J. Eur. Acad. Dermatol. Venereol..

[34] Laurita R (2017). Cold atmospheric plasma treatment of infected skin tissue: Evaluation of sterility, viability, and integrity. IEEE Trans. Radiat. Plasma Med. Sci..

[35] Gay-Mimbrera J (2016). Clinical and biological principles of cold atmospheric plasma application in skin cancer. Adv. Ther..

[36] Gherardi M, Tonini R, Colombo V (2018). Plasma in dentistry: Brief history and current status. Trends Biotechnol..

[37] Kim JH, Lee MA, Han GJ, Cho BH (2014). Plasma in dentistry: A review of basic concepts and applications in dentistry. Acta Odontol. Scand..

[38] Cha S, Park YS (2014). Plasma in dentistry. Clin. Plasma Med..

[39] Kim GC (2013). Dental applications of low-temperature nonthermal plasmas. Plasma Process. Polym..

[40] Xiong Z, Roe J, Grammer TC, Graves DB (2016). Plasma treatment of onychomycosis. Plasma Process. Polym..

[41] Bulson JM (2020). Non-thermal atmospheric plasma treatment of onychomycosis in an in vitro human nail model. Mycoses.

[42] de Morais Gouvêa Lima G (2022). Cold atmospheric pressure plasma is effective against *P. gingivalis* (HW24D-1) mature biofilms and non-genotoxic to oral cells. Appl. Sci..

[43] Park JH (2015). A comparative study for the inactivation of multidrug resistance bacteria using dielectric barrier discharge and nano-second pulsed plasma. Sci. Rep..

[44] Lunov O (2014). Cell death induced by ozone and various non-thermal plasmas: Therapeutic perspectives and limitations. Sci. Rep..

[45] Daeschlein G (2014). In vitro susceptibility of multidrug resistant skin and wound pathogens against low temperature atmospheric pressure plasma jet (APPJ) and dielectric barrier discharge plasma (DBD). Plasma Process. Polym..

[46] Nicol MJ (2020). Antibacterial effects of low-temperature plasma generated by atmospheric-pressure plasma jet are mediated by reactive oxygen species. Sci. Rep..

[47] Graves DB (2017). Mechanisms of plasma medicine: Coupling plasma physics, biochemistry, and biology. IEEE Trans. Radiat. Plasma Med. Sci..

[48] Bourke P, Ziuzina D, Han L, Cullen PJ, Gilmore BF (2017). Microbiological interactions with cold plasma. J. Appl. Microbiol..

[49] Gilmore BF (2018). Cold plasmas for biofilm control: Opportunities and challenges. Trends Biotechnol..

[50] Zimmermann JL (2012). Test for bacterial resistance build-up against plasma treatment. New J. Phys..

[51] Cohen ML (1992). Epidemiology of drug resistance: Implications for a post-antimicrobial era. Science.

[52] Tomasz A (1994). Multiple-antibiotic-resistant pathogenic bacteria. New Eng. J. Med..

[53] Livermore DM (2000). Antibiotic resistance in Staphylococci. Int. J. Antimicrob. Agents.

[54] Lowy FD (2003). Antimicrobial resistance: The example of *Staphylococcus aureus*. J. Clin. Investig..

[55] Levy SB, Marshall B (2004). Antibacterial resistance worldwide: Causes, challenges and responses. Nat. Med..

[56] Eichenberger EM, Thaden JT (2019). Epidemiology and mechanisms of resistance of extensively drug resistant gram-negative bacteria. Antibiotics.

[57] Bombaywala S, Mandpe A, Paliy S, Kumar S (2021). Antibiotic resistance in the environment: A critical insight on its occurrence, fate, and eco-toxicity. Environ. Sci. Pollut. Res..

[58] Zhang H, Zhang C, Han Q (2023). Mechanisms of bacterial inhibition and tolerance around cold atmospheric plasma. Appl. Microbiol. Biotechnol..

[59] Graves DB (2012). The emerging role of reactive oxygen and nitrogen species in redox biology and some implications for plasma applications to medicine and biology. J. Phys. D Appl. Phys..

[60] Dharini M, Jaspin S, Mahendran R (2023). Cold plasma reactive species: Generation, properties, and interaction with food biomolecules. Food Chem..

[61] Machala Z, Tarabová B, Sersenová D, Janda M, Hensel K (2018). Chemical and antibacterial effects of plasma activated water: Correlation with gaseous and aqueous reactive oxygen and nitrogen species, plasma sources and air flow conditions. J. Phys. D Appl. Phys..

[62] Sakiyama Y, Graves DB, Chang HW, Shimizu T, Morfill GE (2012). Plasma chemistry model of surface microdischarge in humid air and dynamics of reactive neutral species. J. Phys. D Appl. Phys..

[63] Peng Y (2022). Kinetic study of key species and reactions of atmospheric pressure pulsed corona discharge in humid air. Plasma Sci. Technol..

[64] Kossyi IA, Kostinsky AY, Matveyev AA, Silakov VP (1992). Kinetic scheme of the non-equilibrium discharge in nitrogen–oxygen mixtures. Plasma Sources Sci. Technol..

[65] Ehlbeck J (2010). Low temperature atmospheric pressure plasma sources for microbial decontamination. J. Phys. D Appl. Phys..

[66] Bruggeman PJ, Iza F, Brandenburg R (2017). Foundations of atmospheric pressure non-equilibrium plasmas. Plasma Sources Sci. Technol..

[67] Tendero C, Tixier C, Tristant P, Desmaison J, Leprince P (2006). Atmospheric pressure plasmas: A review. Spectrochim. Acta B Atom. Spectrosc..

[68] Laroussi M, Akan T (2007). Arc-free atmospheric pressure cold plasma jets: A review. Plasma Process. Polym..

[69] Winter J, Brandenburg R, Weltmann KD (2015). Atmospheric pressure plasma jets: An overview of devices and new directions. Plasma Sources Sci. Technol..

[70] Korbut AN, Kelman VA, Zhmenyak YV, Klenovskii MS (2014). Emission properties of an atmospheric-pressure helium plasma jet generated by a barrier discharge. Opt. Spectrosc..

[71] Brandenburg R (2017). Dielectric barrier discharges: Progress on plasma sources and on the understanding of regimes and single filaments. Plasma Sources Sci. Technol..

[72] Lu X, Laroussi M (2005). Optimization of ultraviolet emission and chemical species generation from a pulsed dielectric barrier discharge at atmospheric pressure. J. Appl. Phys..

[73] Thiyagarajan M, Alexeff I, Parameswaran S, Beebe S (2005). Atmospheric pressure resistive barrier cold plasma for biological decontamination. IEEE Trans. Plasma Sci..

[74] Fridman A, Chirokov A, Gutsol A (2005). Non-thermal atmospheric pressure discharges. J. Phys. D Appl. Phys..

[75] Khun J, Scholtz V, Hozák P, Fitl P, Julák J (2018). Various DC-driven point-to-plain discharges as non-thermal plasma sources and their bactericidal effects. Plasma Sources Sci. Technol..

[76] Fridman A, Nester S, Kennedy LA, Saveliev A, Mutaf-Yardimci O (1999). Gliding arc gas discharge. Prog. Energy Combust. Sci..

[77] Coulombe S, Léveillé V, Yonson S, Leask RL (2006). Miniature atmospheric pressure glow discharge torch (APGD-t) for local biomedical applications. Pure Appl. Chem..

[78] Ni Y, Lynch MJ, Modic M, Whalley RD, Walsh JL (2016). A solar powered handheld plasma source for microbial decontamination applications. J. Phys. D Appl. Phys..

[79] Walsh JL, Kong MG (2011). Portable nanosecond pulsed air plasma jet. Appl. Phys. Lett..

[80] Pei X, Liu J, Xian Y, Lu X (2014). A battery-operated atmospheric-pressure plasma wand for biomedical applications. J. Phys. D Appl. Phys..

[81] Parkey J (2015). A battery powered, portable, and self-contained non-thermal helium plasma jet device for point-of-injury burn wound treatment. Plasma Process. Polym..

[82] Thiyagarajan M (2013). A portable atmospheric air plasma device for biomedical treatment applications. J. Med. Dev..

[83] Ho KN, Chaijaruwanich A (2018). Thermal characteristics of helical coiled heat exchanger with graphene-deionized water on waste heat recovery of combustion stack gas. CMU J. Nat. Sci..

[84] do Nascimento F (2023). A low cost, flexible atmospheric pressure plasma jet device with good antimicrobial efficiency. IEEE Trans. Radiat. Plasma Med. Sci..

[85] Khun J (2021). Non-thermal plasma sources based on cometary and point-to-ring discharges. Molecules.

[86] Scholtz V, Julák J (2010). The, “cometary” discharge, a possible new type of DC electric discharge in air at atmospheric pressure, and its bactericidal properties. J. Phys. Conf. Ser..

[87] Scholtz V, Julák J (2010). Plasma jetlike point-to-point electrical discharge in air and its bactericidal properties. IEEE Trans. Plasma Sci..

[88] Julák J, Scholtz V (2013). Decontamination of human skin by low-temperature plasma produced by cometary discharge. Clin. Plasma Med..

[89] Paldrychová M (2019). Effect of non-thermal plasma on AHL-dependent QS systems and biofilm formation in *Pseudomonas aeruginosa*: Difference between non-hospital and clinical isolates. AIP Adv..

[90] Scholtz V, Julák J, Kříha V (2010). The microbicidal effect of low-temperature plasma generated by corona discharge: Comparison of various microorganisms on an agar surface or in aqueous suspension. Plasma Process. Polym..

[91] Scholtz V, Kvasničková E, Julák J (2013). Microbial inactivation by electric discharge with metallic grid. Acta Phys. Pol. A.

[92] Švarcová M, Julák J, Hubka V, Soušková H, Scholtz V (2015). Treatment of a superficial mycosis by low-temperature plasma: A case report. Prague Med. Rep..

[93] Vaňková E (2019). Prevention of biofilm re-development on Ti–6Al–4V alloy by cometary discharge with a metallic grid. Contrib. Plasma Phys..

[94] *Guide to Creating a Portable Non-thermal Plasma Source*. https://ufmt.vscht.cz/pns.

[95] do Nascimento F (2023). Plasma electrode dielectric barrier discharge: Development, characterization and preliminary assessment for large surface decontamination. Plasma Chem. Plasma Process..

[96] Andrés CMC, de Lastra JMP, Juan CA, Plou FJ, Pérez-Lebeña E (2023). Superoxide anion chemistry—Its role at the core of the innate immunity. Int. J. Mol. Sci..

[97] Lam PL (2020). The role of reactive oxygen species in the biological activity of antimicrobial agents: An updated mini review. Chem. Biol. Interact..

[98] Robinson M (1961). Movement of air in the electric wind of the corona discharge. AIEE Trans..

[99] Moreau E (2007). Airflow control by non-thermal plasma actuators. J. Phys. D Appl. Phys..

[100] Li L, Lee SJ, Kim W, Kim D (2015). An empirical model for ionic wind generation by a needle-to-cylinder dc corona discharge. J. Electrostat..

[101] Sigmond RS, Lagstad IH (1993). Mass and species transport in corona discharges. High Temp. Chem. Proces..

[102] Moreau E, Benard N, Lan-Sun-Luk JD, Chabriat JP (2013). Electrohydrodynamic force produced by a wire-to-cylinder DC corona discharge in air at atmospheric pressure. J. Phys. D Appl. Phys..

[103] Rickard M, Dunn-Rankin D, Weinberg F, Carleton F (2006). Maximizing ion-driven gas flows. J. Electrostat..

[104] Deng XL, Nikiforov AY, Vanraes P, Leys C (2013). Direct current plasma jet at atmospheric pressure operating in nitrogen and air. J. Appl. Phys..

[105] Akishev Y, Grushin M, Karalnik V, Petryakov A, Trushkin N (2010). Non-equilibrium constricted DC glow discharge in N_2_ flow at atmospheric pressure: Stable and unstable regimes. J. Phys. D Appl. Phys..

[106] Callebaut T, Kochetov I, Akishev Y, Napartovich A, Leys C (2004). Numerical simulation and experimental study of the corona and glow regime of a negative pin-to-plate discharge in flowing ambient air. Plasma Sources Sci. Technol..

[107] Gilmore FR, Laher RR, Espy PJ (1992). Franck–Condon factors, *r*-centroids, electronic transition moments, and Einstein coefficients for many nitrogen and oxygen band systems. J. Phys. Chem. Ref. Data.

[108] Guerra V, Loureiro J, Sa PA (2001). Role played by the N_2_(A^3^Σ_*u*_^+^) metastable in stationary N_2_ and N_2_–O_2_ discharges. J. Phys. D Appl. Phys..

[109] Lu X (2016). Reactive species in non-equilibrium atmospheric-pressure plasmas: Generation, transport, and biological effects. Phys. Rep..

[110] van Gaens W, Bogaerts A (2014). Reaction pathways of biomedically active species in an Ar plasma jet. Plasma Sources Sci. Technol..

[111] Pontiga, F. and Castellanos, A. Nitrogen oxides generation induced by negative corona discharge in N_2_ + O_2_ mixtures. In *IEEE Conf. Electr. Insul. Dielectr. Phenom.* 264–267 (2006).

[112] Liu F, Wang W, Zheng W, Wang Y (2008). Investigation of spatially resolved spectra of OH and N_2_^+^ in N_2_ and H_2_O mixture wire-plate positive pulsed streamer discharge. Spectrochim. Acta A Mol. Biomol. Spectrosc..

[113] Herron JT (1999). Evaluated chemical kinetics data for reactions of N(^2^D), N(^2^P), and N_2_(A^3^Σ_*u*_^+^) in the gas phase. J. Phys. Chem. Ref. Data.

[114] Ono R (2016). Optical diagnostics of reactive species in atmospheric-pressure nonthermal plasma. J. Phys. D Appl. Phys..

[115] Murakami T, Niemi K, Gans T, O'Connell D, Graham WG (2014). Afterglow chemistry of atmospheric-pressure helium–oxygen plasmas with humid air impurity. Plasma Sources Sci. Technol..

[116] Huang L (2021). Bactericidal effect of surface plasma under different discharge modes. Phys. Plasmas.

[117] Brisset JL, Hnatiuc E (2012). Peroxynitrite: A re-examination of the chemical properties of non-thermal discharges burning in air over aqueous solutions. Plasma Chem. Plasma Process..

[118] Pavlovich MJ, Clark DS, Graves DB (2014). Quantification of air plasma chemistry for surface disinfection. Plasma Sources Sci. Technol..

[119] van Bokhorst-van de Veen H (2015). Inactivation of chemical and heat-resistant spores of *Bacillus* and *Geobacillus* by nitrogen cold atmospheric plasma evokes distinct changes in morphology and integrity of spores. Food Microbiol..

[120] Kolb JF (2012). Cold DC-operated air plasma jet for the inactivation of infectious microorganisms. IEEE Trans. Plasma Sci..

[121] Kučerová K, Machala Z, Hensel K (2020). Transient spark discharge generated in various N_2_/O_2_ gas mixtures: Reactive species in the gas and water and their antibacterial effects. Plasma Chem. Plasma Process..

[122] Wang J (2018). Antimicrobial mechanism and the effect of atmospheric pressure N_2_ plasma jet on the regeneration capacity of *Staphylococcus aureus* biofilm. Biofouling.

[123] Lukes P, Dolezalova E, Sisrova I, Clupek M (2014). Aqueous-phase chemistry and bactericidal effects from an air discharge plasma in contact with water: Evidence for the formation of peroxynitrite through a pseudo-second-order post-discharge reaction of H_2_O_2_ and HNO_2_. Plasma Sources Sci. Technol..

[124] Sharma M, Hudson JB (2008). Ozone gas is an effective and practical antibacterial agent. Am. J. Infect. Control.

[125] Wang X (2022). Analysis of bactericidal effect of three medical ozonation dosage forms on multidrug-resistant bacteria from burn patients. Infect. Drug Resist..

[126] Eto H, Ono Y, Ogino A, Nagatsu M (2008). Low-temperature sterilization of wrapped materials using flexible sheet-type dielectric barrier discharge. Appl. Phys. Lett..

[127] Park JS, Sung BJ, Yoon KS, Jeong CS (2016). The bactericidal effect of an ionizer under low concentration of ozone. BMC Microbiol..

[128] Dobrynin D, Friedman G, Fridman A, Starikovskiy A (2011). Inactivation of bacteria using DC corona discharge: Role of ions and humidity. New J. Phys..

[129] Douat C, Hübner S, Engeln R, Benedikt J (2016). Production of nitric/nitrous oxide by an atmospheric pressure plasma jet. Plasma Sources Sci. Technol..

[130] Yehia A, Mizuno A (2013). Ozone generation by negative direct current corona discharges in dry air fed coaxial wire-cylinder reactors. J. Appl. Phys..

[131] Janda M, Martišovitš V, Hensel K, Machala Z (2016). Generation of antimicrobial NOx by atmospheric air transient spark discharge. Plasma Chem. Plasma Process..

[132] Kogelschatz U (2003). Dielectric-barrier discharges: Their history, discharge physics, and industrial applications. Plasma Chem. Plasma Process..

[133] Liu Z (2017). Production of simplex RNS and ROS by nanosecond pulse N_2_/O_2_ plasma jets with homogeneous shielding gas for inducing myeloma cell apoptosis. J. Phys. D Appl. Phys..

[134] Oehmigen K (2010). The role of acidification for antimicrobial activity of atmospheric pressure plasma in liquids. Plasma Process. Polym..

[135] Bartis EAJ, Knoll AJ, Luan P, Seog J, Oehrlein GS (2016). On the interaction of cold atmospheric pressure plasma with surfaces of bio-molecules and model polymers. Plasma Chem. Plasma Process..

[136] Zhou R (2020). Plasma-activated water: Generation, origin of reactive species and biological applications. J. Phys. D Appl. Phys..

[137] Liu DX (2016). Aqueous reactive species induced by a surface air discharge: Heterogeneous mass transfer and liquid chemistry pathways. Sci. Rep..

[138] Mai-Prochnow A (2021). Interactions of plasma-activated water with biofilms: Inactivation, dispersal effects and mechanisms of action. NPJ Biofilms Microbiomes.

[139] Timoshkin IV (2012). Bactericidal effect of corona discharges in atmospheric air. IEEE Trans. Plasma Sci..

[140] Rainey FA (2005). Extensive diversity of ionizing-radiation-resistant bacteria recovered from Sonoran Desert soil and description of nine new species of the genus *Deinococcus* obtained from a single soil sample. Appl. Environ. Microbiol..

[141] Agapov AA, Kulbachinskiy A (2015). Mechanisms of stress resistance and gene regulation in the radioresistant bacterium *Deinococcus radiodurans*. Biochemistry (Moscow).

[142] Slade D, Radman M (2011). Oxidative stress resistance in *Deinococcus radiodurans*. Microbiol. Mol. Biol. Rev..

[143] Mattimore V, Battista JR (1996). Radioresistance of *Deinococcus radiodurans*: Functions necessary to survive ionizing radiation are also necessary to survive prolonged desiccation. J. Bacteriol..

